# Emergence of heartbeat frailty in advanced age I: perspectives from life-long EKG recordings in adult mice

**DOI:** 10.1007/s11357-022-00605-4

**Published:** 2022-06-27

**Authors:** Jack M. Moen, Christopher H. Morrell, Michael G. Matt, Ismayil Ahmet, Syevda Tagirova, Moran Davoodi, Michael Petr, Shaquille Charles, Rafael de Cabo, Yael Yaniv, Edward G. Lakatta

**Affiliations:** 1 Laboratory of Cardiovascular Science, National Institute On Aging, National Institutes of Health, Baltimore, MD USA; 2 Present Address: Department of Cellular and Molecular Pharmacology, UCSF, San Francisco, CA USA; 3 Present Address: Pediatric Residency Program, Cincinnati Children’s Hospital Medical Center, Cincinnati, OH USA; 4 Biomedical Engineering Faculty, Technion-IIT, Haifa, Israel; 5 Laboratory of Experimental Gerontology Intramural Research Program, National Institute On Aging, National Institutes of Health, Baltimore, MD USA; 6 Present Address: Center for Healthy Aging, University of Copenhagen, Copenhagen, Denmark

**Keywords:** Heart rate variability, Sinoatrial node, Longitudinal aging, Autonomic nervous system

## Abstract

The combined influences of sinoatrial nodal (SAN) pacemaker cell automaticity and its response to autonomic input determine the heart’s beating interval variability and mean beating rate. To determine the intrinsic SAN and autonomic signatures buried within EKG RR interval time series change in advanced age, we measured RR interval variability before and during double autonomic blockade at 3-month intervals from 6 months of age until the end of life in long-lived (those that achieved the total cohort median life span of 24 months and beyond) C57/BL6 mice. Prior to 21 months of age, time-dependent changes in intrinsic RR interval variability and mean RR interval were relatively minor. Between 21 and 30 months of age, however, marked changes emerged in intrinsic SAN RR interval variability signatures, pointing to a reduction in the kinetics of pacemaker clock mechanisms, leading to reduced synchronization of molecular functions within and among SAN cells. This loss of high-frequency signal processing within intrinsic SAN signatures resulted in a marked increase in the mean intrinsic RR interval. The impact of autonomic signatures on RR interval variability were net sympathetic and partially compensated for the reduced kinetics of the intrinsic SAN RR interval variability signatures, and partially, but not completely, shifted the EKG RR time series intervals to a more youthful pattern. Cross-sectional analyses of other subsets of C57/BL6 ages indicated that at or beyond the median life span of our longitudinal cohort, noncardiac, constitutional, whole-body frailty was increased, energetic efficiency was reduced, and the respiratory exchange ratio increased. We interpret the progressive reduction in kinetics in intrinsic SAN RR interval variability signatures in this context of whole-body frailty beyond 21 months of age to be a manifestation of “heartbeat frailty.”

**Supplementary Information:**

The online version contains supplementary material available at 10.1007/s11357-022-00605-4.

## Introduction

Although, by convention, the number of heartbeats per minute in vivo is casually referred to as the heart rate (HR), the heart’s pacemaker is not a metronome, and analyses of successive RR intervals within EKG time series detect substantial beat-to-beat interval variability. Beat-to-beat RR interval variability means that the subcellular, cell-wide, and intercellular mechanisms that underlie sinoatrial nodal (SAN) impulse generation never achieve equilibrium [1, 2], largely, in part, because the neuro-visceral autonomic axis continually conducts impulses at ms to s time scales to and from the heart and other viscera via parasympathetic and sympathetic nerves. Beat-to-beat afferent signals (both neuronal and mechanical) originating within the heart signal to other parts of the heart and back out to the spinal cord, brain stem, and higher CNS structures, which in turn elicit autonomic reflexes via efferent input of the central nervous system to the heart [3]. This autonomic input modulates the heart’s beating rate and rhythm by impacting the rate and rhythm of spontaneous action potential firing generated by mechanisms intrinsic to SAN pacemaker cells. In other terms, continuous interactions on a beat-to-beat basis between the autonomic nervous system (autonomic signatures) and intrinsic SAN mechanisms (SAN signatures) lead to both short term (ms-s) and long-range (s-min) RR interval variability within an EKG time series from which the mean RR interval (heart rate) is derived [1]. In the presence of autonomic blockade, heartbeats occur without having autonomic input. In this context, beat-to-beat variability of intrinsic base physiologic functions within and among SAN cells determine the RR interval variability.

Following the discovery that a coupled-clock system regulated pacemaker cell function [2, 4], it was demonstrated that mechanisms that underlie beat-to-beat variability in AP firing intervals in single isolated SAN pacemaker cells lie in the extent of coupling between calcium clocks and surface membrane clocks that are intrinsic to SAN pacemaker cells [5]. Combined in vitro, ex vivo, and in vivo studies indicated that beat-to-beat variability in RR intervals within an EKG time series informs on these mechanisms that govern beat-to-beat variability in SAN cells. Thus, information concerning the kinetics of mechanisms that drive the coupled system intrinsic to SAN pacemaker cells could be assessed via analyses of time, frequency, and nonlinear RR variability signatures buried within an EKG RR interval time series.

A reduction in RR intervals’ variability (reduced heart rate variability, HRV) within an EKG time series has been observed in cardiac disease states, e.g., heart failure. It portends increased mortality in the context of cardiac diseases, e.g., post-myocardial infarction [6]. Heart rate variability also becomes reduced in the context of advancing age in humans and animal models [7, 8]. Although the resting heart rate in humans changes very little beyond young adulthood, the mean intrinsic RR interval, measured during dual sympathetic and parasympathetic autonomic blockade, begins to decrease substantially between young adulthood and middle age [4] but has not been studied in humans of older ages.

Because autonomic input to the SAN masks age-associated intrinsic SAN dysfunction, intrinsic RR interval variability measured in the presence of double autonomic blockade is likely to inform on the overall health status of the SAN in the absence of confounding effects of autonomic input. Changes in intrinsic RR interval variability may be the earliest markers of SAN failure, aka “sick sinus syndrome,” manifested as sinus bradycardia, SAN impulse pauses, and irregularity of rhythms of RR intervals within EKG time series that result from SAN cell dysfunction that increases exponentially with advanced age, often requiring electronic pacemaker implantation that ultimately progresses to SAN failure in advanced age. Detailed analyses of changes in RR interval variability provide insight into subcellular mechanisms.

The neuro-visceral autonomic axis continually conducts impulses at ms to s time scales to and from the heart and other viscera via parasympathetic and sympathetic nerves. Beat-to-beat afferent signals (both neuronal and mechanical) originating within the heart signal to other parts of the heart and back out to the spinal cord, brain stem, and higher CNS structures, which in turn elicit autonomic reflexes via efferent input of the central nervous system to the heart [3]. This autonomic input modulates the heart’s beating rate and rhythm by impacting the rate and rhythm of spontaneous action potential firing generated by mechanisms intrinsic to SAN pacemaker cells. Although, by convention, the number of heartbeats per minute is casually referred to as the in vivo HR, analyses of successive RR intervals within an EKG time series detect substantial beat-to-beat interval variability, indicating that subcellular and cell-wide mechanisms that underlie SAN impulse generation never achieve equilibrium [5, 9].

The combined influences of SAN pacemaker cell automaticity and its response to autonomic input determine the heart’s beating interval variability and mean beating rate. When the heart beats in the absence of autonomic input, intrinsic base physiologic functions of SAN cells determine beat-to-beat interval variability from which the mean beating rate is calculated. SAN failure, aka “sick sinus syndrome,” manifests as sinus bradycardia, SAN impulse pauses, and irregular rhythms of RR intervals within EKG time series resulting from SAN cell dysfunction increase exponentially with advanced often requiring electronic pacemaker implantation. Although the resting heart rate in humans changes very little beyond young adulthood, the mean *intrinsic* RR interval, measured during dual sympathetic and parasympathetic autonomic blockade, begins to decrease substantially between young adulthood and middle age [10] and has been shown also to be reduced in a small heterogenous sample in older persons, but has not been studied systematically in adults of older ages [11]. Because autonomic input to the SAN masks age-associated intrinsic dysfunction, intrinsic RR interval variability is likely to inform on the overall health status of the SAN in the absence of confounding effects from autonomic input, and changes in RR interval variability may be the earliest markers of SAN dysfunction that ultimately progresses to SAN failure in advanced age. In short, *intrinsic* RR interval variability reflects the status of functions intrinsic to SAN cells that regulate the rate and rhythm of impulse emergence from the SAN.

The aging mouse manifests HR and heartbeat interval variability abnormalities that recapitulate many of those described in humans [8, 12–15]. *Cross-sectional* studies that compare average data in different mice that have survived to different ages indicate that the intrinsic heartbeat interval variability and mean intrinsic HR are relatively constant between young adulthood and middle age but become substantially altered later in life [8]. The increased sympathetic input to the SAN of older mice helps to reduce intrinsic heartbeat interval variability towards that of mice at younger ages. Other cross-sectional studies have associated changes in SAN automaticity in older mice with whole-body frailty [14, 16]. However, because these cross-sectional studies lack *mouse*-*specific*, *longitudinal perspectives*, they are plagued by marked heterogeneity of results in mice of older ages when whole-body frailty becomes manifest [17–19]. Longitudinal data on heart rhythm frailty in mice (or in humans) are mainly lacking because the longitudinal study design requires repeated measures of HRV that are implemented over a large part of the life course. The most important measures of cardiac frailty can be extended to mice, enabling the identification of SAN longevity markers in animals that live to or beyond the median life span.

We reasoned that changes in longitudinal measurements of intrinsic heartbeat interval variability in a given mouse will inform on the signature of late-life functional deterioration within and among SAN pacemaker cells. This functional deterioration leads to progressive changes in RR interval variability that increase the mean intrinsic RR interval in that mouse. To this end, we designed and implemented a longitudinal study that assessed heartbeat interval variability repeatedly in mice at 3-month intervals, beginning at 6 months of age and continuing to the end of life. We recorded EKG time series at each age prior to (basal state) and during double autonomic blockade (intrinsic state) throughout the entire life span months in a cohort of 56 C57/BL6 black mice. This design not only permitted the elucidation of how the signature of mechanisms that underlie intrinsic SAN cell pacemaker function deteriorates over the life course, but also enabled elucidation of the autonomic signature, i.e., a set of measurements for the differences between mean basal and mean intrinsic state parameters measured at each age in a given mouse. Rates at which autonomic signatures change over the life course inform on the rates at which they compensate for intrinsic SAN deterioration as these signatures progress towards frailty. The unique nature of this experimental design enabled the unique comparisons of the deterioration of intrinsic SAN function and compensatory autonomic signatures of heartbeat interval variability in advanced age to more traditional noncardiac whole-body frailty indices and measurements of movement-based energetic efficiency and energy substrate utilization.

## Methods

### Electrocardiograms during anesthesia

Fifty-eight C57/BL6 mice were obtained from Charles River labs. All studies were performed in accordance with the Guide for the Care and Use of Laboratory 102 Animals published by the National Institutes of Health (NIH Publication no. 85–23, revised 1996). Experimental protocols were approved by the Animal Care and Use Committee of the National Institutes 104 of Health (protocol #441-LCS-2016).

Under light anesthesia with 2% isoflurane (Baxter Corp) at 0.2 ml/min, electrode needles were inserted under the skin, and the mice were allowed to adapt to these conditions for 20 min. EKG recordings were made using Power Lab 6, with signals obtained at a sampling rate of 10,000 Hz. A three-lead electrocardiogram was then recorded for 50 min: 10 min prior to and 40 min after intraperitoneal injection of a saline solution containing atropine (75 µg/ml) and propranolol (150 µg/ml). EKG time series were recorded in each mouse at 3-month intervals, starting at 6 months of age and continuing for the entire life span of each mouse. Saline solutions were given based on body weight, at a ratio of 400 µl for 30-g mouse. EKG recordings were made in the same room at constant temperature (25 °C) and humidity (44%). During recordings, a heat lamp was positioned at a constant distance (approximately 25 cm) from the mouse’s body to prevent heat loss.

### Electrocardiograms via telemetry

Studies were implemented in compliance with the Guide for the Care and Use of Laboratory Animals by the National Institutes of Health. The animal study protocol was approved by the Animal Care and Use Committee of the National Institute on Aging (ASP 441-LCS-2019). Mice were kept on a standard 12-h light–dark cycle, single-housed with corn pop bedding, and free access to food and water. Young (3–4 months of age, *n* = 10) and old (24 months of age, *n* = 10) male C57/BL6 mice were implanted with telemetry devices (ETA-F10, Data Sciences International, St. Paul, MN). After a 2-week recovery period, mice were subjected to EKG recordings. A 30-min baseline EKG at a sampling rate of 1 kHz was recorded. A double autonomic blockade consisting of atropine (0.5 mg/kg) and propranolol (1 mg/kg) diluted in saline (6.6 ml/kg) was administered via an intraperitoneal (i.p.) injection, and the EKG recording continued for another 60 min. The maximum responses to double autonomic blockade typically occurred within 20 ~ 30 min of administration and were confirmed by real-time observations of heart rate changes.

Average EKG RR intervals were assessed from 2- to 4-min segments in 24–30 M old awake (*n* = 6) and anesthetized (*n* = 6) mice during double autonomic blockade by PhysioZoo Open Access Platform [20], and the mean of average RR intervals, percent change, and delta RR were calculated. Statistical significance of differences between means were tested by Student’s *t* test. A value of *p* < 0.05 was considered statistically significant.

### EKG RR interval time series analyses

Measures of RR interval variability parameters assessed in time, frequency, nonlinear, and fragmentation domains prior to and during dual autonomic blockade inform on the contributions of intrinsic and autonomic signatures of RR interval variability. EKG time series of RR intervals were analyzed using PhysioZoo [20]. A mouse preset with rodent T waves was used to obtain a block average. Initially, 1-min averages were obtained from the 10 min prior to injection and 40 min following injection. A block average was made from the 1-min averages for the set of basal recordings and recordings in the presence of pharmacological autonomic blockade. Recordings of a single mouse were removed from the entire dataset, being an outlier with a resting heart rate > 700 bpm, and were removed from the entire dataset prior to analysis.

Heart rate variability analysis also employed PhysioZoo. ECGs were first analyzed to identify segments that fit strict selection criteria. This was mainly dependent on whether the heart rate was stationary for a sufficiently long period of time. Stationarity was determined based on the absence of linear trends, a high ectopic number, or a nonstable heart rate. Segments that met these eligibility criteria were included in the subset of tracings used for heart rate variability (for specific *N* numbers refer to Table S1). Generally, a 1.5–2-min segment was selected, containing between 512 and 1024 intervals. Instead, larger subsets were obtained for frequency domain analysis. The software was set up to auto analyze segments based on the length and segmented into similar sizes. Thus, our data was reported on files clipped to 512 intervals. After this, we applied an automatic ectopic removal correction [20] in which any values outside of two standard deviations were removed.

The control RR peaks were named “basal” and “intrinsic” RR peaks were named “abk.” Each recording included a basal segment (e.g., no drug intervention), followed by a segment collected under the influence of atropine and propranolol. Heartbeat interval segment with a duration of 3 [min] was used. This window was chosen according to the limit of the very low-frequency (VLF) band [20, 21], which is 0.0056–0.152 [Hz] and corresponds to a range of around 6 [s] to 3 [min]. The time windows were nonoverlapping. The low-frequency (LF) and high-frequency (HF) bands were also set according to Behar et al. [20, 21] as 0.152–1.24 [Hz] and 1.24–5 [Hz], respectively. We have excluded the first 2 [min] of the “abk” segments to avoid transients.

When analyzing the heartbeat interval data, we have chosen one of the three interval-filtering methods provided by the PhysioZoo platform [20, 21] based on physiological heart rate. The range was defined as 0.05–0.24 [s], corresponding to specific heart rate ranges of 250–1200 [bpm]. This means that every interval that was not in that range was excluded, and the remaining intervals were interpolated.

In addition to the transient and interval-filtering mentioned above, we defined a new method to check whether the mice went through a complete dual autonomic blockade at each age. The basal recordings of mice who have failed to pass the criteria of the autonomic blockade were also excluded. Our method was set as a physiological criterion. We chose to examine the following:

1. Whether the HF peak was diminished after double blockade injection.
2. If the average total power per window was lower after double blockade injection.

The average was used since the “abk” records are mostly about four times longer in duration than basal.

The criteria were calculated in the HF band using the AR (autoregressive) model with the order of 30 and are described as follows:

In the basal state, at every time window of every mouse, we calculated the frequency where the PSD (power spectral density) gets its peak (maximum value), and we denote it as f_peak. In every time window, there is a small deviation of this frequency, so the representative peak frequency was chosen to be the average, i.e.,

$${\overline{\mathrm f}}_{\mathrm{peak}}=\frac1{{\mathrm N}_{\mathrm b}}\sum_{\mathrm j=1}^{{\mathrm N}_{\mathrm b}}{\mathrm f}_{\mathrm{peak}}(\mathrm j)$$

where ${N}_{b}$ is the number of time windows in the basal mode for the current mouse. To examine how prominent this peak was, we normalized the PSD at the average frequency peak as follows:

$${\overline{\mathrm H}}_{\mathrm b}\left({\overline{\mathrm f}}_{\mathrm{peak}}\right)=\frac1{{\mathrm S}_{\mathrm b}{\mathrm N}_{\mathrm b}}\sum_{\mathrm j=1}^{{\mathrm N}_{\mathrm b}}{\mathrm H}_{\mathrm{bj}}\left({\overline{\mathrm f}}_{\mathrm{peak}}\right)$$

where ${H}_{bj}$ is the PSD of the *j*th window and ${S}_{b}$ is the total power along with all the windows, i.e.,

$${\mathrm S}_{\mathrm b}=\sum_{\mathrm j=1}^{{\mathrm N}_{\mathrm b}}{\mathrm{total}}_{\mathrm{power}}\left(\mathrm j\right)$$

with the average total power being:

$${\overline{\mathrm S}}_{\mathrm b}=\frac{{\mathrm S}_{\mathrm b}}{{\mathrm N}_{\mathrm b}}$$

In the “abk” adequate records, we used the same average frequency as in basal and calculated the PSD value there, but the normalization was applied using the total power and number of windows of the “abk” mode. Thus:

$${\overline{\mathrm H}}_{\mathrm k}\left({\overline{\mathrm f}}_{\mathrm{peak}}\right)=\frac1{{\mathrm S}_{\mathrm k}{\mathrm N}_{\mathrm k}}\sum_{\mathrm j=1}^{{\mathrm N}_{\mathrm k}}{\mathrm H}_{\mathrm{kj}}\left({\overline{\mathrm f}}_{\mathrm{peak}}\right)$$

and

$${\overline{\mathrm S}}_{\mathrm k}=\frac{{\mathrm S}_{\mathrm k}}{{\mathrm N}_{\mathrm k}}$$

where $k$ is used for denoting “abk.”

The blockade process was defined as successful if at least one of the following was satisfied:

1. The PSD peak at “abk” mode was less than 0.5 of its parallel value in basal mode:
  $$\frac{{\overline{\mathrm H}}_{\mathrm k}\left({\overline{\mathrm f}}_{\mathrm{peak}}\right)}{{\overline{\mathrm H}}_{\mathrm b}\left({\overline{\mathrm f}}_{\mathrm{peak}}\right)}<0.5$$
2. The average total power per window in “abk” decreased by at least 30%; thus,
  $$\frac{{\overline{\mathrm S}}_{\mathrm k}}{{\overline{\mathrm S}}_{\mathrm b}}<0.7$$

We found that the majority of mice across all of the ages were successfully gone through autonomic blockade according to these criteria. Here is a table that summarizes the number of mice that were not disqualified and their percentage with respect to the number of mice in every age.

**Table:** 

6	9	12	15	18	21	24	27	30
*n* = 56	*n* = 53	*n* = 54	*n* = 53	*n* = 46	*n* = 42	*n* = 28	*n* = 17	*n* = 3
96.55%	91.37%	94.73%	96.36%	90.19%	95.45%	93.33%	100%	75%

### HRV parameters measured

Terms are derived from Behar et al. [20].

**Table:** 

Measures	Units	Definition
Time domain		
SDNN	[ms]	Standard deviation of NN interval duration. It primarily reflects autonomic influence on HRV but it can tell us about both short- and long-term variability depending on the window size. “Short” window correlates with short-term variability. Calculated as: $\sqrt{\frac{1}{N-1}\sum_{i=0}^{N-1}{(NN\left({t}_{i}\right)-\overline{NN })}^{2}}$
pNN5	[%]	Percent of NN interval differences greater than 5 ms. Calculated as: $\frac{100}{N-1}\sum_{i=1}^{N-1}{I}_{i}^{5}$ where ${I}_{i}^{5}$ is the indicator function. 5 is set to 5 for mice
PIP	[%]	Percentage of inflection points in the NN interval time series, defined as the percentage of zero-crossing points in the increment time series. A point, ${t}_{{N}_{i}}$, is defined as an inflection point if $\Delta {NN}_{i} *{NN}_{i+1}\le 0.$
IALS	[n.u]	Inverse average length of the acceleration/deceleration segments. An acceleration/deceleration segment is a sequence of NN intervals between consecutive inflection points for which the difference between two NN intervals is smaller/larger than 0 respectively
PSS	[%]	Percentage of short segments. This quantity is defined as the complement of the percentage of NN intervals in acceleration and deceleration segments with three or more NN intervals
PAS	[%]	The percentage of NN intervals in alternation segments. An alternation segment is a sequence of at least four NN intervals, for which heart rate acceleration changes sign every beat. Such sequences follow an “ABAB” pattern, where “A” and “B” represent increments of the opposite sign
Frequency		
Total power	[ms^2^]	Estimated total power spectral density
VLF	[ms^2^]	Power in the very low-frequency band. This measure is correlated with the SAN functionality (intrinsic nervous system) and thus affects mostly on long-term variability
LF	[ms^2^]	Power in the low-frequency band. One of the physiological interpretations for this band is that it reflects the baroreceptor reflex frequency response and thus relates to the short-term variability but it is not quite clear yet
HF	[ms^2^]	Power in the high-frequency band. The power in the HF band is attributed to vagal (parasympathetic) stimulation of the heart and the HF peak has been shown to correspond to the parasympathetic modulation at a frequency synchronous with the breathing rate, a phenomenon known as respiratory sinus arrhythmia (RSA)
VLF %	[%]	Low-frequency power in normalized units. Calculated as:$100*LF/(Total power)$
LF %	[%]	Low-frequency power in normalized units. Calculated as:$100*LF/(Total power-VLF)$
HF %	[%]	High-frequency power in normalized units. Calculated as:$100*HF/(Total power-VLF)$
LF/HF	[n.u]	Low-frequency band to high-frequency band power ratio. Mostly used to estimate the ration between the sympathetic and parasympathetic activity respectively
*β*	[n.u]	Slope of the linear interpolation of the spectrum in a log–log scale for frequencies below the upper bound of the VLF band. Since the spectrum also exhibits power-law scaling and, in fact, $S(f)\propto {\tau f}^{\beta }$, we can use $\beta$ as a measure of irregularity of the signal
Nonlinear		
SampEn	[n.u]	Sample entropy is also a measure of irregularity since entropy increases once the signal is more irregular. It does this by looking at the probability that two similar sequences of length $m$ in the signal are also similar for length *m* + 1. High entropy means that that signal is less predicted and might tell us about its complexity
SD1	[ms]	NN interval standard deviation along the first principle axis of the Poincaré plot. SD1 informs on short-range correlations among RR intervals within the EKG time series
SD2	[ms]	NN interval standard deviation along the second principle axis of the Poincaré plot. SD2 informs on long-range correlations among intervals
SD1:SD2	[ms]	SD1:SD2 informs on the nonlinearity of the correlations of RR intervals within a given EKG time series
DFA	[n.u.]	DFA slope coefficients *α*_1_ and *α*_2_ together inform on nonlinearity of short-range DFA (slope coefficient *α*_1_) and long-range (DFA slope coefficient *α*_2_) RR interval correlations within an EKG time series

### Statistics

All statistical analyses were implemented in R 3.2.3 [22] using RStudio [23].

### Mixed ANOVA analyses

To test for age-associated differences in mean response, age was set as a factor assuming that recording intervals were approximately the same, and a mixed-effect ANOVA was conducted (Table S2).

### Linear mixed-effect statistical analyses

A linear mixed-effect (LME) model (lmerTest) was used to evaluate the effect of time following the initial measurement at 6 months of age on basal and intrinsic RR interval parameters and autonomic index HR and HRV parameters.

### Estimating mouse-specific rates of change in RR interval variability

The average age trajectory is displayed using a loess smooth curve [24]. For many EKG time series parameters, the trajectory over the age span is highly nonlinear, exhibiting a sharp change around 21 months of age. A simple polynomial cannot adequately model this sharp change. Consequently, when needed, the trajectories are modeled in two parts: ages 6–21 months and 21 to 30 months. To accommodate the curvature in the trajectories within each of these age spans, a quadratic model in age is adopted, when needed. In this case, the full LME model becomes:

$${\mathrm y}_{\mathrm{ij}}=\left({\mathrm\beta}_0+{\mathrm b}_{\mathrm i0}\right)+\left({\mathrm\beta}_1+{\mathrm b}_{\mathrm i1}\right)\mathrm{Age}+\left({\mathrm\beta}_2+{\mathrm b}_{\mathrm i2}\right)\mathrm{Age}^2+{\mathrm\varepsilon}_{\mathrm{ij}}$$

LME models contain both *fixed* and *random* terms. The *β*s are the regression parameters for the *fixed-effect* variables, while *b*_*i*0_, *b*_*i*1_, and *b*_*i*2_ are the corresponding random effects. *ε*_*i*_ is the usual error term. The fixed-effect terms of LME models are the usual regression-type explanatory variables and their parameter estimates provide estimates of *average* rates of change in the response variable for changes in the associated variable. However, the random effect terms of LME models allow for variability in initial value and trajectories among the mice. To obtain mouse-specific rates of change, age must now be considered as a numerical variable, and the trajectory over the age span must be appropriately modeled. The random intercept effect (*b*_*i*0_) allows for variability in individual mouse intercepts or starting points, while the additional random effects, *b*_*i*1_ and *b*_*i*2_, allow for the linear and quadratic terms to vary among the animals.

In the full model, the rate of change in the response for individual mouse *i* is the derivative of the model function with respect to age and is given by:

$${\mathrm{Rate}}_{\mathrm i}=\left({\mathrm\beta}_1+{\mathrm b}_{\mathrm i1}\right)+2\times\left({\mathrm\beta}_2+{\mathrm b}_{\mathrm i2}\right)\times\mathrm{Age}$$

where the *β*s and *b*_*i*_s are replaced by the estimates obtained from the data. When terms are eliminated from the model, the appropriate remaining terms from the final model are used to obtain the mouse-specific rates of change. For many variables, the data could not accommodate the random effect for Age^2^. Consequently, in these cases, this random effect was removed from the model.

### Frailty assessment

To compare the onset and severity of heart beat frailty derived from our longitudinal EKG analyses, we conducted a cross-sectional study to assess traditional, noncardiac-specific, organism-wide frailty in mice of advanced age. We used a validated mouse frailty index consisting of noncardiac factors [17]. Variability among mice in heart functions that change at an older age have been shown to correlate better with this frailty index score than with chronologic age [25]. The frailty index was administered to a subset of older aged awake mice. A mouse frailty assessment form was administered in parallel [13]. The assessment was performed in parallel by two different investigators in order to deter bias. The scores from each assessment were summed from the 31 parametric observations to calculate the mouse’s frailty score. The scores determined by each investigator were averaged to generate the final frailty score.

### Energetic efficiency

Work was measured as the distance traveled per unit of time and energetic efficiency. Energetic efficiency, defined as kinetic work performed/oxygen consumption/unit time, was assessed cross-sectionally in another subset of older mice. Oxygen consumption was measured through an enclosed treadmill coined as “metabolic treadmill,” where the mouse is subjected to run at its age group’s average gait speed determined by over-ground walking analysis in earlier TSE MotoRater assessments. To ensure familiarity, mice were acclimated on the metabolic treadmills (Columbus Instruments International, Columbus, OH) at 5 m/min for 30 min the day before testing. The following day, mice ran at the previously defined age-matched natural walking gait speed for 45 min [26]. The external motivators included electric shock from an electrified metal grid near the moving belt to entice mice to run. Mice unwilling or incapable of running after being shocked 5 consecutive times within a few seconds met the criterion for exhaustion, and the testing ended.

## Results

### Longitudinal cohort longevity

**Fig. 1:**
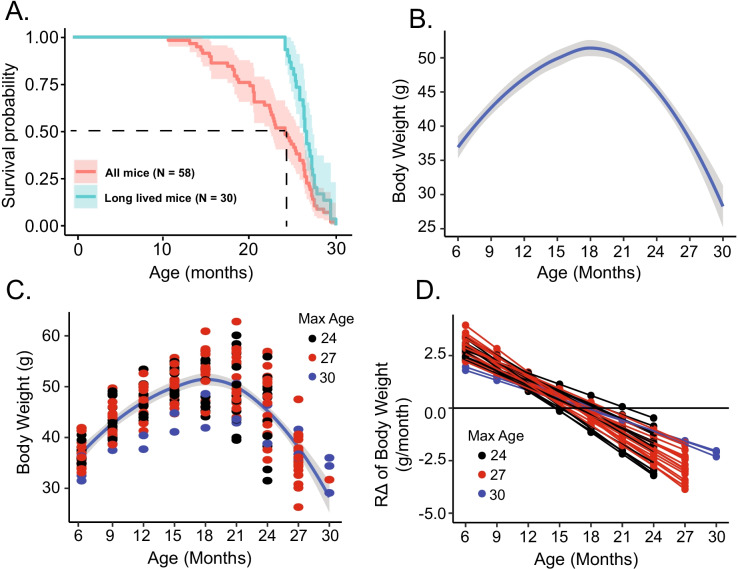
**A** Kaplan–Meier survival curves for entire cohort of mice (*n* = 58) and for long-lived subset of mice (*n* = 30). Average loess smooth curve with 95% confidence interval for body weight (**B**) with mouse-specific values (**C**). **D** Mouse-specific *R*Δ of body weight

The Kaplan–Meier survival curve of our entire C57/BL6 cohort is shown in Fig. 1A. Our study’s median life span of the total C57/BL6 cohort (*n* = 58) was approximately 24 months (Fig. 1). Because the main focus of our study was to identify how HR and HRV become altered in advanced age, we analyzed the life-long (6–30-month) EKG time series of each mouse in the basal and intrinsic states. We took 30 months of age as our mouse cohort’s maximum life span because only 3 of 58 (5.2% of the entire cohort) survived to this age. We focused on long-lived (LL) mice (*n* = 29), which we defined as those that achieved the median cohort life span of 24 months. Thus, the number of LL mice that survived from 6 to 24 months of age is constant at all ages and that beyond 24 months of age, the number of surviving mice becomes reduced as aging progresses, i.e., *n* = 17, and 3 at 27 and 30 months respectively (Table S1).

The survival curve for the LL subset of our total mouse cohort is also shown in Fig. 1A. Note its relative rectangularity compared to the survival curve of the entire cohort and its steep rate of decline relative to that of the entire cohort. The average life span of LL mice was 27.4 months (834 days or 119 weeks and 1 day) and relative to the entire cohort, and LL mice at 24 months of age had already achieved about 75% of the mouse maximum life span.

### Longitudinal changes in body weight

Loss of body weight is essential in noncardiac frailty and may be an important biomarker in longevity [27]. Body weight in the long-lived mouse cohort changed in a nonlinear manner, increasing significantly with age up to 18 months and significantly declining after 21 months until the end of life (Fig. 1B), with the variability in mouse-specific *R*Δ increasing beyond 18 months (Fig. 1C). Considering body weight loss as a sign of frailty, the earliest signs of organismal frailty appear to emerge as early as 18 months (Fig. 1B and C).

### Signatures within EKG time series rhythms measured longitudinally over the adult life course

The kinetics and degrees of molecular synchronization and their modulation by autonomic input determine rhythms buried within an EKG time series. We assessed the EKG time series RR interval variability patterns in the time, frequency, nonlinear, and fragmentation domains. Means and RR interval variability data for all measured parameters at each age are listed in Table S1. The corresponding mixed-effect ANOVA analyses of these data are presented in Table S2.

### SAN signature in the time domain

**Fig. 2:**
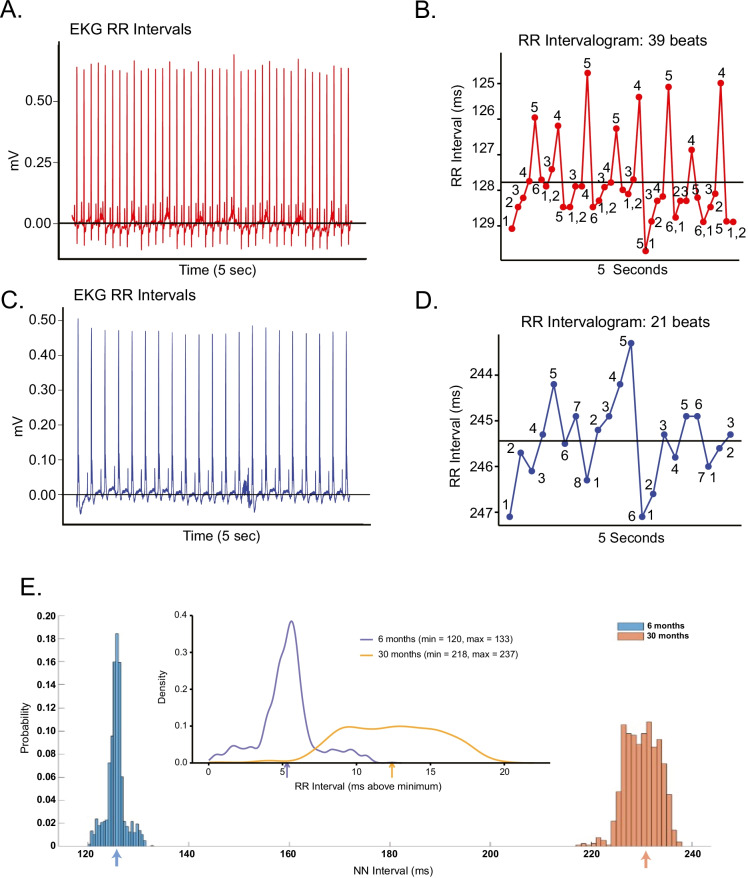
EKG time series measured in the presence of dual autonomic blockade at 6 months (**A**) and 30 months (**C**) in the same mouse. Corresponding RR intervelograms at 6 months (**B**) and 30 months (**D**) measured in the same mouse. **E** Distributions of intrinsic RR intervals at 6 and 30 months for the same mouse (ZA1730) as in panels **A**–**D**; arrows indicate the mean RR interval at both ages. **E** (Inset) RR interval distributions aligned at the minimum RR interval at each age

Intrinsic rhythms buried within an EKG RR interval time series are assessed during the dual autonomic blockade. Representative EKG RR interval time series recorded during the autonomic blockade of a LL mouse at 6 and 30 months of age are shown in Fig. 2A and C. RR interval variability (Fig. 2B and D) confirms that the SAN does not function as a metronome [28]: the time at which the next beat is generated is based on its memory of intrinsic mechanisms affected by the rate and rhythm of near term prior beats [5]; in other terms, as the heart beats in real time, it has no a priori knowledge of its *mean* beating rate during an RR interval time series. Thus, a *mean* RR interval within a time series does not exist in real time but is calculated post hoc from RR intervals (solid lines in Fig. 2B and D) as the sum of individual (and variable) RR intervals in an EKG time series that occur over a fixed period. Note that at 6 months of age (Fig. 2A and B), 39 beats were generated during a 5-s time interval, whereas at 30 months of age in the same mouse (Fig. 2C and D), only 21 beats were generated during a 5-s interval; importantly, the interbeat intervals are not fixed but vary (Fig. 2B and D). The mean RR interval of an EKG time series does not capture this exquisite RR interval variability but assumes that all intervals are equal.

Nonconstant interval variability among RR intervals within an EKG time series generates short- and long-range correlations, nonlinearity components, and variable frequency distributions of RR intervals within the time series [29]. These RR interval rhythms buried within an EKG time series inform on the beat-to-beat variability in the kinetics of molecular mechanisms and the extent to which they are synchronized within and among cells [5]. The age-associated shifts in the RR interval variability pattern in the intrinsic state for the same representative mouse measured over a 2-year period is striking (Fig. 2B compared to Fig. 2D, right panels); note the regular recurrent pattern of each of the six to seven numbered beats at 6 months of age. Repetition of this pattern over several beats requires utilizing memory functions within and among pacemaker cells that reside within SAN tissue. This memory-dependent pattern of successive RR intervals becomes markedly distorted in the same mouse at 30 months (Fig. 2D), suggesting that advanced age is associated with an impairment of youthful memory functions within and among pacemaker cells. Figure 2E shows the distributions of intrinsic RR intervals in the time domain (during autonomic blockade) for the same LL mouse at 6 and 30 months of age depicted in Fig. 2A–D. Note that not only is the RR interval (ms) distribution shifted to longer intervals at 30 months than at 6 months of age, causing the mean RR interval to increase dramatically, but the synchronization of RR intervals within the distributions becomes markedly reduced at 30 months than at 6 months, illustrated more clearly when the distributions are normalized to their minimum value (Fig. 2E inset). This suggests that the loss of memory required to reproduce recurrent patterns is associated with reduced synchronization of molecular functions within pacemaker cells or reduced cell-to-cell synchronization.

The percentage of long RR intervals in an EKG time series provides another perspective on the recurring patterns of RR intervals. In humans, this calculation is pNN50, e.g., the percentage of NN intervals that exceed 50 ms, whereas in mouse, the corresponding interval is 5 ms (pNN5) [20]. The average pNN5 increased from 6.28 ± 13.97 at 18 months to 12.45 ± 11.72 at 27 months (*p* < 0.002), and the *R*Δ of pNN5 for individual mice throughout the life course is shown in Fig. S1. Intrinsic pNN5 was correlated with mean RR both at 6 and 24 months of age (Fig. S1, panels B and C).

**Fig. 3:**
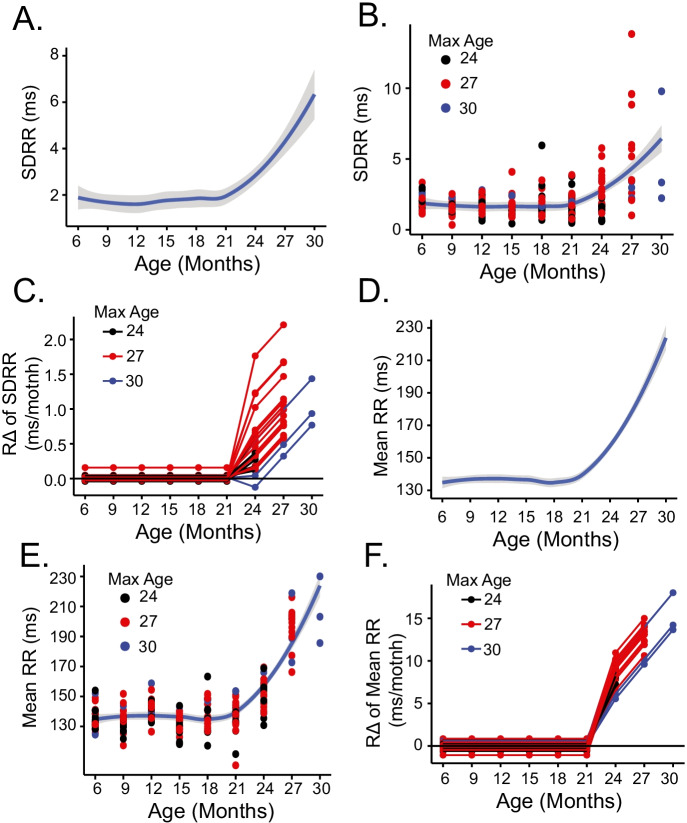
**A** Average loess smooth curve with 95% confidence interval of intrinsic SD of the mean RR (SDRR). **B** Mouse-spcific intrinsic SDRR in long-lived mice. **C** Mouse-specific *R*Δ of SDRR in long-lived mice. **D** Average loess smooth curve with 95% confidence interval for intrinsic mean RR. **E** Mouse-spcific intrinsic mean RR in long-lived mice. **F** Mouse-specific *R*Δ for intrinsic mean RR in long-lived mice

The SD of the RR intervals (SDRR) can also be calculated from the distribution of RR intervals. The *basal* SDRR has generally been attributed to the magnitude of parasympathetic input to the SAN [30], but the *intrinsic* SDRR measured during double autonomic blockade reflects beat-to-beat variability about the mechanisms *intrinsic* to SAN cells [31]. The average SDRR of all LL mice as a function of age is shown in Fig. 3A, and the SDRR of all individual mice are shown in Fig. 3B. Our experimental design permitted the calculation of mouse-specific rates of change (*R*Δ) measured in the presence of double autonomic blockade, providing aggregate RR interval data. In addition to informing on the rate at which SAN signatures within an EKG time series change during a given mouse’s life course (i.e., mouse-specific rates), the *R*Δ also provides insight into the rates at which inter-mouse-to-mouse variability of rates at which RR interval signatures change throughout the life course. *R*Δ of intrinsic SDRR individual LL mice are illustrated in Fig. 3C, the most significant increase occurring between 21 and 24 months of age. Mouse-specific intrinsic RR intervals of LL mice between the ages of 6 and 24–30 months are shown in Fig. 3E. The *R*Δ of intrinsic SDRR in individual LL mice (Fig. 3F) occurs between 21 and 24 months.

**Fig. 4:**
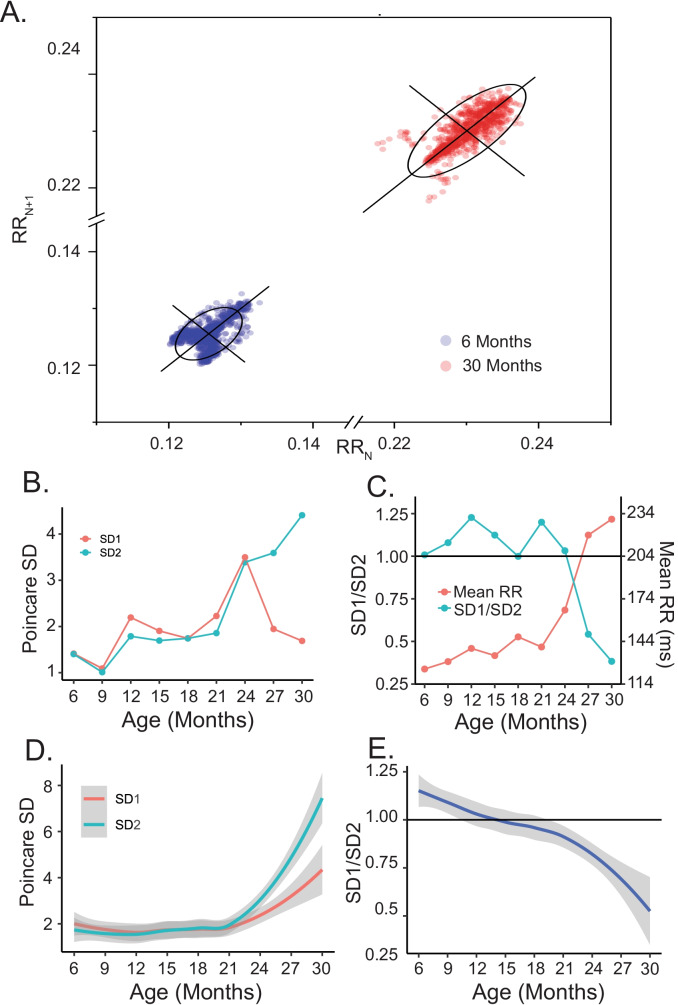
**A** Poincaré plot of intrinsic RR interval data at 6 and 30 months for the same mouse in Fig. 2. **B** Intrinsic SD1 and SD2 for the same mouse. **C** Intrinsic SD1/SD2 and mean RR for the same mouse. **D** Average loess smooth curves with 95% confidence intervals of intrinsic SD1 and SD2. **E** Average loess smooth curves with 95% confidence intervals of intrinsic SD1/SD2

The Poincaré diagram is a convenient, informative method to visualize distributions of successive RR intervals within a time series that underlie the mean RR interval that relates to each other in the time domain [32]. In a Poincaré diagram, a given RR interval (*N*) is plotted against the next RR interval (*N* + 1). Figure 4A shows the Poincaré diagram of the LL mouse RR intervals presented in Fig. 2. The points within the Poincaré plot can be fit to an ellipsoid, with the spread of the data measured as the SD1 or SD2; the Poincaré SD1 informs on short-range correlations among RR intervals within the EKG time series, whereas SD2 informs on long-range correlations among intervals [32]. The intersection of SD1 and SD2 at the center of the ellipse is the mean RR interval, and the SD1:SD2 informs on the nonlinearity of the correlations of RR intervals within a given EKG time series [32].

Intrinsic SD1, SD2, SD1:SD2, and the mean intrinsic RR interval throughout the entire adult life span of the LL mouse in Figs. 2 and 4A are shown in Fig. 4B and C. Note how the SD1 declines while SD2 increases beyond 21 months, creating progressive reductions in SD1:SD2. The average intrinsic SD1, SD2, and SD1:SD2 mean intrinsic RR intervals of all the LL cohort over its entire life span are shown in Fig. 4D and E. As in the indexed mouse in Figs. 2 and 3A on average, SD1, SD2, and the mean RR interval of LL mice markedly increased beyond 21 months of age, with the SD2 increasing to a much greater extent than SD1 (Fig. 4D), reflecting nonlinearity (SD1:SD2) of short- and long-range RR interval correlations as age increases beyond 21 months (Fig. 4E).

The mean RR interval within a time series, as noted, is a post hoc average of all RR intervals that occur within a given time series; the shift from shorter to longer RR intervals between 21 and 30 months (Fig. 2) causes the mean RR interval to increase over this age range (Fig. 3D). The average intrinsic mean RR of all LL mice increases markedly beyond 21 months (Fig. 3D) (note that the number of mice remains constant between 6 and 24 months of age and decreases between 24 and 30 months of age, becoming markedly reduced between 27 and month 30 months). The intrinsic RR interval of the individual LL mice between the ages of 6 and 24–30 months is shown in Fig. 3E. The *R*Δ of the mean intrinsic RR interval of all LL mice is illustrated in Fig. 3F.

### Signature of SAN RR interval rhythms in the frequency domain

**Fig. 5:**
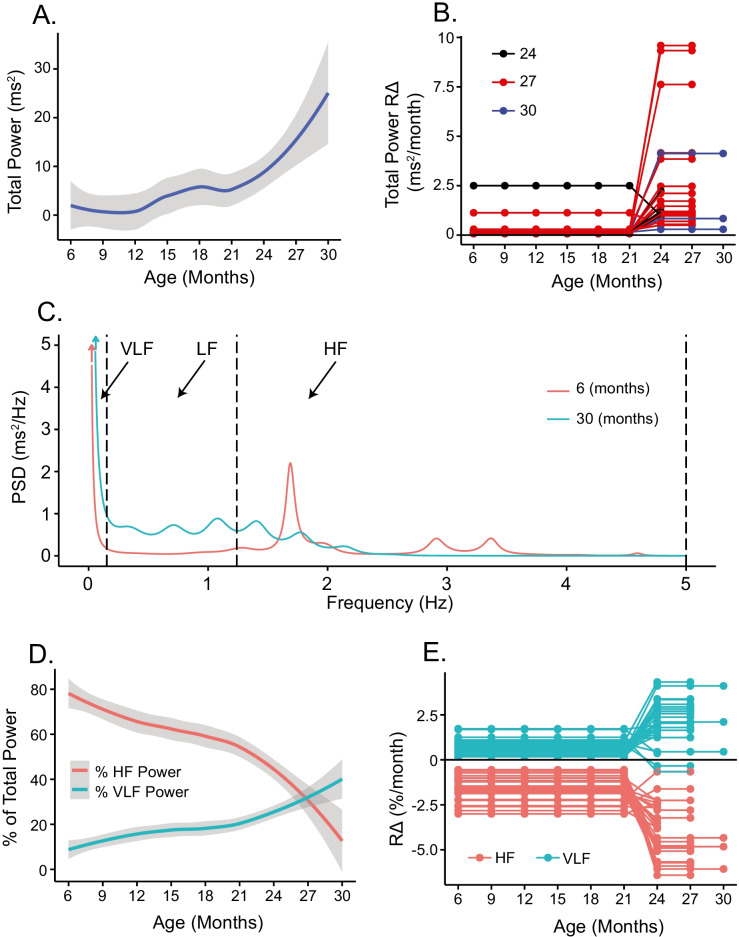
**A** Average loess smooth curve with 95% confidence interval of intrinsic total power (PSD). **B** Mouse-specific rates of change of intrinsic total power (PSD) in long-lived mice. **C** Intrinsic frequency domain data at 6 and 30 months for mouse ZA1730. **D** Average loess smooth curves with 95% confidence intervals of intrinsic VLF and HF. **E** Mouse-specific rates of change of intrinsic VLF and HF in long-lived mice

Fast Fourier transforms provide power spectral densities (PSD) of RR intervals within an EKG interval time series, identifying different frequencies of hidden rhythms that are not evident in EKG time domain analyses [33]. A greater total power informs on greater complexity, i.e., less coherence and more variability among RR intervals within the RR interval time series. In the absence of autonomic blockade, increased total power has been linked to vagal influences on RR interval rhythms, but during autonomic blockade, an increase in total power informs on the deterioration of aforementioned mechanisms intrinsic to and among SAN cells that determine when the next impulse will emerge. Between 6 and 21 months, the mean intrinsic total power of the LL cohort did not vary significantly (Fig. 5A, Table S2), but, beyond 21 months of age, the mean intrinsic total power *increased* by about twofold, similar to *R*Δ for SDRR (Fig. 4F). *R*Δ of both total power and SDRR varied substantially among LL mice between 21 and 30 months (Fig. 5B).

The total power can be partitioned into regions that differ in their approximate frequencies, including high-frequency (HF), low-frequency (LF), and very low-frequency (VLF) power components (Fig. 5C, Table S1). During dual autonomic blockade, relative HF power informs on the ability of SAN cells to generate short RR intervals. Power spectra of RR intervals within the EKG time series at 6 months and 30 months of age in the same long-lived mouse depicted in Figs. 1, 2, 3, and 4 are shown in Fig. 5C. Note the striking reduction in relative HF power between 6 and 30 months of age, while LF and VLF power markedly increases, informing on a reduction in the number of short RR intervals over this age range. Average values of intrinsic VLF power components normalized to total power in all mice increased modestly between 6 and 21 months (Fig. 5D, Table S2). The contribution of intrinsic HF power to intrinsic total power declined by about five-fold between 6 and 30 months in LL mice (Fig. 5D, Table S2). VLF power increased exponentially while HF power plummeted dramatically. Note that beyond 21 months, the intrinsic %VLF power increased exponentially as intrinsic %HF power plummeted (Fig. 5D, Table S2). Beyond 27 months, the *intrinsic* %VLF power exceeded intrinsic %HF power. Note that the mouse-specific *R*Δ for %HF and %VLF power are mirror images of each other (Fig. 5E). Reductions in intrinsic HF and increases in VLF power, in part at least, reflect the inability to generate short RR intervals (Fig. 2B and D) and age-associated increases in short (SD1) and long (SD2) RR interval correlations within the EKG RR interval time series (Fig. 4).

### Other SAN signatures in the nonlinear domain

Detrended fluctuation analysis (DFA) describes correlations between successive NN intervals. Like the SD1:SD2, the DFA slope coefficients *α*_1_ and *α*_2_ together inform on nonlinearity of short-range DFA (slope coefficient *α*_1_) and long-range (DFA slope coefficient *α*_2_) RR interval correlations within an EKG time series [29]. Examples of DFA analyses at 6 and 30 months in the same long-lived mouse depicted in Figs. 1, 2, and 3 are shown in Figure S1A. The calculated slope coefficients, *α*_1_ and *α*_2_, throughout the entire life course of this mouse are shown in Fig. S3 and Tables S1 and S2. The average intrinsic *α*_1_ and *α*_2_ of the entire LL cohort also increased throughout the life span, accelerating sharply (nearly doubling) between 21 and 30 months (Fig. S3C, Tables S1 and S2). Mouse-specific *R*Δ for *α*_1_ and *α*_2_ for all LL mice are shown in Fig. S3D. The increase in *α*_2_ (reflecting long-range correlations) can be visualized in the RR intervelograms in Fig. 2B and D.

### SAN RR interval fragmentation signatures

RR interval fragmentation analyses that attempt to quantify the erratic beating of the SAN by detecting small, aperiodic long-range fluctuations in RR intervals are an expansion of the aforementioned RR interval variability analyses [34]. RR interval fragmentation parameters assessed include percentage of inflection points (PIP), or points at which the first RR difference changes sign; the percentage of short RR segments (PSS); the inverse of the average RR segment length (IALS); and the percentage of alternating RR segments (PAS). A larger value of each parameter denotes a more fragmented RR interval structure within the RR interval time series.

**Fig. 6:**
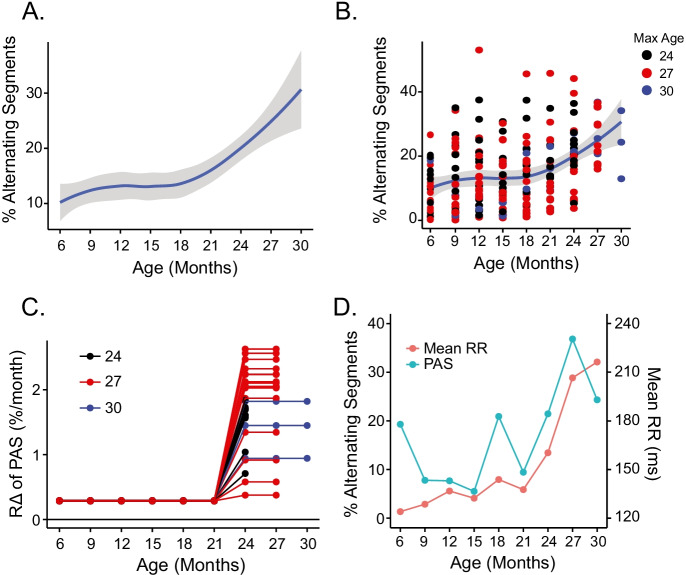
**A** Average loess smooth curve with 95% confidence interval of intrinsic PAS. **B** Mouse-specific rates of change of intrinsic PAS in long-lived mice. **C** Trajectories of intrinsic PAS in mouse ZA1730. **D** Change in % alternating segments and mean RR across life span

Changes in PIP, IALS, and PSS in our LL cohort resembled each other across the mouse life span (Fig. S4): (a) at ages less than 21 months, intrinsic IALS PIP and PSS displayed slight but statistically significant age-associated trends; (b) beyond 21 months, neither mean intrinsic PIP nor IALS of LL mice changed significantly with age (Table S2), but intrinsic PSS did increase beyond 21 months (Fig. S4, Table S2). In contrast to these RR interval fragmentation signatures, the average PAS in LL mice increased sharply beyond 21 months of age (Fig. 6A). The mouse-specific absolute values and *R*Δ of PAS for the LL mice is seen in Fig. 6B and C. The mean RR and PAS across the entire age span for the same mouse highlighted in Figs. 1, 2, 3, 4, and 5 are shown in Fig. 6D. Note the parallelism between increases in PAS and mean RR interval changes during the latter part of life.

**Fig. 7:**
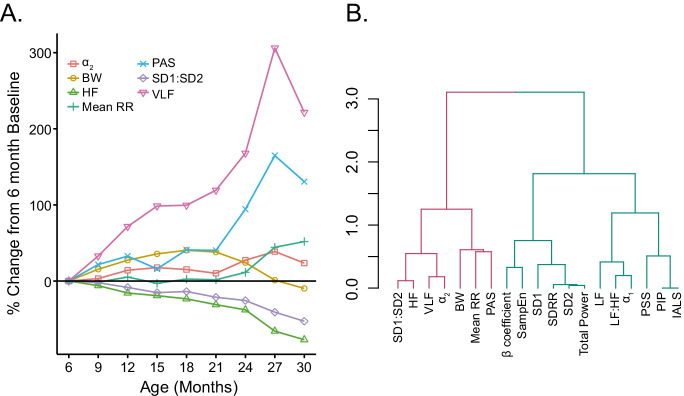
**A** Cluster analysis dendrogram of intrinsic rates of change in the late time period (after 21 months) in long-lived mice. **B** Relative change of intrinsic mean RR and RR interval variability from 6-month baseline

To compare the relative magnitudes of changes in RR interval variability parameters (signature of SAN aging in Table S2 and Fig. S4) that underlie the marked prolongation of the intrinsic age-associated RR interval signatures during advanced age in LL, we normalized parametric values measured at 3-month intervals in each mouse to their respective 6-month values (Fig. 7A). Intrinsic %VLF power increased by about 2.5-fold; in contrast, intrinsic %HF power became reduced by about 20%, and the Poincaré SD1/SD2 was reduced by 50% between 6 and 30 months. PAS increased by 50%, and DFA long-range intrinsic *α*_2_ increased by 25%. Note that beyond 21 months of age, the increase in mean intrinsic RR interval is paralleled by sharp increases in *α*_2_, PAS, and %VLF power with the relative change in %VLF power greater than PAS, greater than *α*_2_; and by sharp reductions in SD1:SD2 and %HF power (beyond 24 months in Fig. 7A). The net result of these intrinsic RR interval variability measures was to produce, on average, a 50% increase in the mean RR interval between 6 and 30 months, with much of the increase occurring, as within the RR interval variability parameters, beyond 21 months.

We next sought to discover the *R*Δ of intrinsic RR interval *variability* signatures of aging in a given mouse that define the rate at which the *mean* intrinsic RR interval increases with age across the later stages of life in that mouse. We performed variable cluster analyses of mouse-specific *R*Δ of all measured RR interval *variability* parameters and the *R*Δ of the *mean* RR interval in each mouse between 21 and 30 months. Figure 7B illustrates the clustering of the *R*Δ of intrinsic RR variability parameters and the mouse-specific *R*Δ of the mean intrinsic RR. Between 21 and 30 months, *R*Δ of intrinsic RR interval *variability* parameters clustered with the *R*Δ of mean intrinsic RR interval in a given mouse (Fig. 7B red cluster) were VLF, HF, *α*_2_, PAS, and SD1:SD2.

Bivariate correlations for mouse-specific *R*Δ of intrinsic RR variability signatures clustered together and with the *R*Δ of the mean RR interval throughout the late-life period (Fig. 7B) are listed in Table 1. *R*Δ of the intrinsic mean RR interval in all of the long-lived individual mice as they age are illustrated in Fig. 3D, with the most significant increase occurring between 21 and 24 months of age. It is important to also note that the *R*Δ of RR interval variability signatures (Tables S1 and S2, Fig. 7B blue cluster) that were excluded from the red cluster in Fig. 7B did *not* correlate with the *R*Δ of the mean intrinsic RR interval between 21 and 30 months of age (Table S3). In other terms, changes in the *R*Δ of variability parameters within the red cluster (Fig. 7B) are sufficient to explain why the *R*Δ of the mean intrinsic RR of LL mice increases in late life.

**Table 1:**
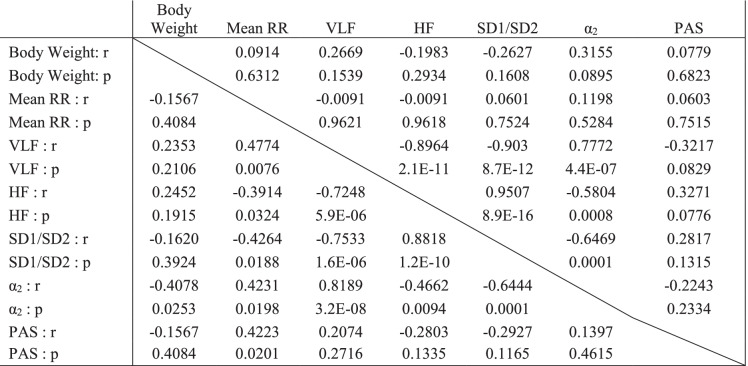
Correlations and *p* values among intrinsic rates of change in long-lived mice

The upper triangle contains the correlations in the early time period (before 21 months) and the lower triangle contains the correlations in the late time period (after 21 months)

The rate at which the mean intrinsic RR increases over time in a given mouse between 21 and 30 months was significantly correlated with concurrent increases in the rates at which *α*_2_ and %VLF power increased and concurrent reductions in *R*Δ of %HF power and SD1:SD2 became reduced. Importantly, this cluster also includes body weight *R*Δ, a component of the frailty assessment index assessed cross-sectionally in another mouse cohort (see below). The rates at which VLF, HF, *α*_2_, and SD1:SD2 RR interval variability signatures underlying the increase in mean intrinsic RR interval during late life were strongly correlated with each other: reductions in the rate of %HF power and SD1:SD2 are positively correlated, and the rates at which %VLF power and α_2_ increase are positively correlated (Fig. S2). Thus, the *R*Δ of %HF and SD1:SD2 concurrently assessed over time inform on the same variability rhythms within the EKG RR interval time series. Similarly, *R*Δ of %VLF power informs on the same rhythms within the EKG time series as does *α*_2_ (Fig. S2). Mouse-specific *R*Δ of %HF and %VLF power were mirror images, and therefore, the rates at which they change are inversely related to each other. The rate at which the mean intrinsic RR increased over time in a given mouse between 21 and 30 months was significantly and positively correlated with concurrent increases in the rates at which %VLF power (or *α*_2_) increased and inversely with the rates at which %HF power (or SD1:SD2) became reduced. Thus, the *R*Δ of %HF and %VLF were inversely related. The inverse correlations of %HF and %VLF power during the late-life course (Fig. S2) reflect the reduction in short RR intervals and increase in long RR intervals in the RR interval frequency distributions in Fig. 2. Importantly, failure to generate short RR (manifest as a reduction in %HF power) intervals informs on reduced synchronization of mechanisms within the coupled-clock system of SAN pacemaker cells.

### The impact of autonomic input of autonomic nervous system signatures on age-associated changes in intrinsic SAN RR interval signatures

Modulation of intrinsic heartbeat intervals imparted by autonomic input to the SAN reflects the combined effects of autonomic neurotransmitter impulses delivered to SAN cells and the responses of these pacemaker cells to that input. Interestingly, the *R*Δ of mean intrinsic RR interval between 18 and 30 months and the signature of the autonomic effect (the difference between the mean intrinsic value and basal value of RR interval parameters in a given mouse) on intrinsic mean RR correlate (*r* = 0.12, *p* = 0.5317 in the early time period and *r* =  − 0.39, *p* = 0.0338 in the late time period) with the change in body weight.

**Fig. 8:**
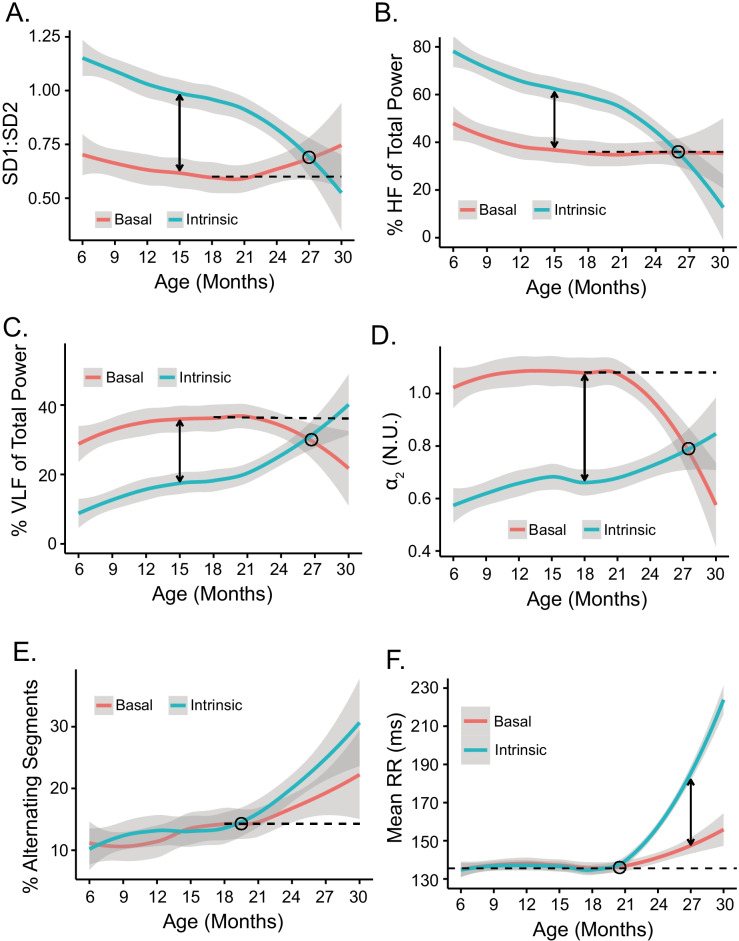
Impact of autonomic signature on intrinsic RR interval variability signatures: **A** SD1:SD2, **B** %HF power, **C** %VLF power, **D** *α*_2_, **E** % alternaing segments %, and on the **F** mean RR interval during the entire life span of the representative mouse featured in Figs. 1,2, 3, 4, 5, and 6

Table S2 and Fig. 8 show how the signatures of autonomic input impact on intrinsic RR interval variability signatures. Note that the difference between intrinsic and basal states of RR interval variability, i.e., autonomic signatures, like that of intrinsic SAN RR interval variability signatures, did not appreciably differ between 6 and 21 months of age (Tables S1 and S2). Between 21 and 30 months of age, the difference between intrinsic and basal state RR interval variability signatures differed markedly within and among each other (Fig. 8A–F, Table S2).

Prior to 21 months of age, SD1:SD2 was substantially less than the average SD1:SD2 of all mice in the intrinsic state (Fig. 8A). Beyond 21 months, the cohort average intrinsic SD1:SD2 began to decline while its basal state counterpart increased, with the average basal and average intrinsic SD1:SD2 converging at 27 months of age. Thus, up until 27 months of age, the effect of autonomic input was to reduce the intrinsic SD1:SD2. The average basal and intrinsic SD1 and SD2 in the three mice surviving up to 30 months of age crossed beyond 27 months, with the intrinsic SD1:SD2 becoming reduced while the basal SD1:SD2 increased. Note that the crossing pattern of basal and intrinsic SD1:SD2 during late life in LL mice is a general feature of other RR variability parameters that correlated with the mean RR intervals in these very old mice, e.g., %HF power (Fig. 8B), %VLF power (Fig. 8C), and DFA slope coefficient *α*_2_ (Fig. 8D).

The effect that autonomic input imparts to %HF power (Fig. 8B) is strikingly similar to its effect on the nonlinearity of short- to long-range RR interval correlations within the time series informed by SD1:SD2 (Fig. 8A). Recall (Fig. S2) that the *R*Δ of intrinsic %HF power and SD1:SD2 in individual mice were positively correlated. The effects of autonomic input on %VLF power (Fig. 8C) and DFA *α*_2_ (Fig. 8D) are also strikingly similar across the entire age span from 6 to 30 months. Recall also (Fig. S2) that (1) the *R*Δ for intrinsic %VLF power and *α*_2_ between 21 and 30 months were positively correlated with each other and to the rates at which the mean RR interval in individual mice changed over this age range and (2) that the *R*Δ for %VLF power and *α*_2_ were inversely correlated with the *R*Δ of %HF power and SD1:SD2 (Fig. S2). Importantly, note in Fig. 8 that the shapes of the mean %VLF power and *α*_2_ (Fig. 8C and D) are also mirror images of those of %HF power and SD1:SD2 (Fig. 8A and B), and note the effects that autonomic signatures impart to intrinsic %HF power and SD1:SD2 are strikingly similar.

PAS was the only RR interval fragmentation index that was not statistically significantly impacted by autonomic input throughout the entire life span (Fig. 8E). Average basal and intrinsic PAS in all LL mice beyond 21 months of age resembled a muted version of the mean intrinsic and basal RR interval (Fig. 8F), but unlike its effect on the mean intrinsic RR interval, the impact of autonomic input on PAS was not significant (Table S2). Although the average basal RR interval of all LL mice did not significantly differ between 6 and 21 months of age, the mean intrinsic RR manifested small but statistically significant changes (Table S2), and beyond 21 months of age, while the average mean intrinsic RR precipitously increased (Fig. 8F), autonomic input had a marked effect of reducing the mean RR interval (Table S2).

### Anesthesia does not affect the autonomic signature

To address the issue of whether anesthesia affects the autonomic signature of the mean RR interval, we inserted telemeters into a subset of LL mice (24–30-month C57/Bl6) to compare the mean RR interval in the presence and absence of autonomic blockade in the awake vs. anesthetized state. As expected, anesthesia increased the mean RR interval (by about 20 ms, Fig. S8), but the increase in the mean basal RR interval was similar to the increase in the mean intrinsic RR interval, such that the autonomic input (the difference between basal and intrinsic) was not significantly impacted by anesthesia (Fig. S8). This suggests that the effects of anesthesia on the mean RR interval in older mice are primarily on components of the intrinsic pacemaker cell excitability mechanisms as shown previously in cardiac ventricular myocytes [35–37].

**Fig. 9:**
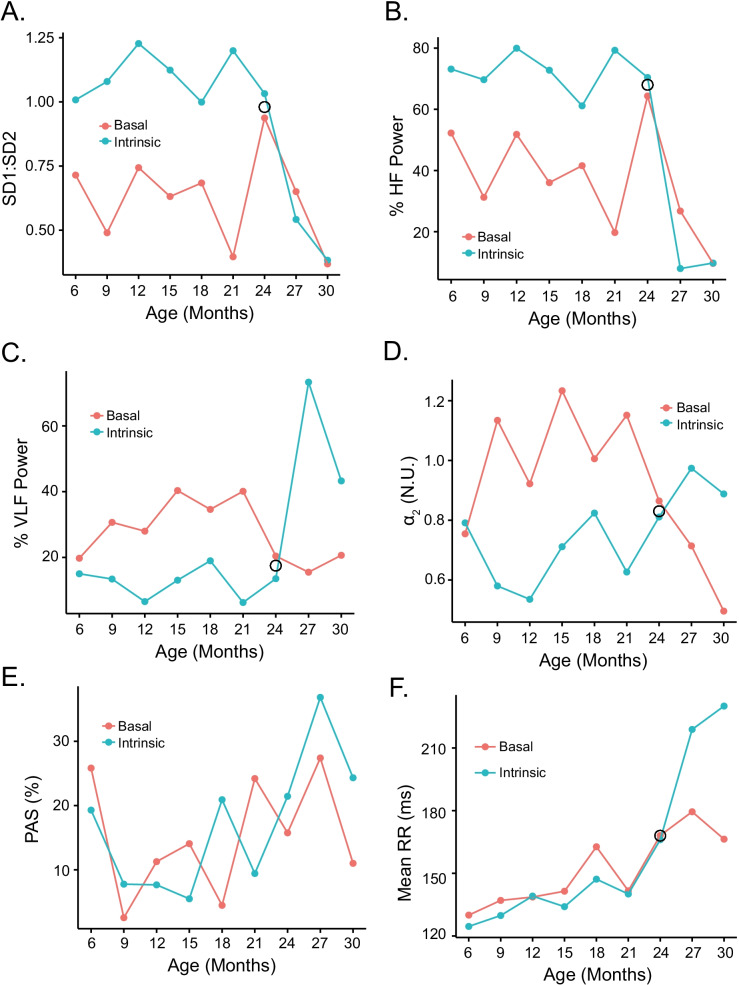
Average loess smooth curves with 95% confidence intervals for basal and intrinsic RR interval variability signatures and on the mean RR interval during the entire life course of all long-lived mice for **A** SD1:SD2, **B** %HF of total power, **C** %VLF of total power, **D** *α*_2_, **E** % alternating sgements, and **F** mean RR

We may deduce that autonomic signatures of changes in RR interval variability measured parameters that correlate with the increase in the mean RR interval between 21 and 30 months of age inform on either failure of autonomic input to the SAN, or the responsiveness of SAN pacemaker cells to this input increases with advanced age in LL mice, or that age-associated changes occur in both mechanisms. Because SAN responsiveness to autonomic neurotransmitters is known to be reduced in advanced age [38, 39], the reduction in mean intrinsic RR interval, i.e., increase in HR, in late life while autonomic input is intact means that autonomic input imparts a *net* sympathetic effect (increased sympathetic or reduced parasympathetic) to autonomic input, because of the directionality of the response (to reduce the RR interval or increase HR) (Fig. 8F). Note, however, that beyond 21 months of age, autonomic input fails to completely restore the mean youthful basal RR interval that prevailed between 6 and 21 months (dashed line in Fig. 8F). This reflects an inability of the net sympathetic autonomic input to overcome the high degree of disorder within the SAN tissue later in life. The impact of autonomic signatures on the intrinsic RR interval signatures over the entire life course of the representative mouse shown in Figs. 1, 2, 3, 4, 5, and 6 are shown in Fig. 9.

Figures S5 and 6 illustrate the average basal and intrinsic RR interval variability parameters of the entire LL cohort having *R*Δ that were *not* significantly correlated with mean RR interval *R*Δ in individual mice, i.e., parameters within the blue cluster in Fig. 7B.

### Differences among individual mice in intrinsic and autonomic RR interval signatures increase in late life

To assess the change in the variability in the rates of change between the early and late periods, the standard deviations of the rates of change were computed at 6 months and at 24 months (Supplementary Table S4). Since the rates come from the same mouse in the two periods, the data is paired and to test if the standard deviations are the same, the Pitman-Morgan test is applied. The table shows that in the intrinsic state, rates of change all increase from the early to the late period; in the basal state, the variability of 5 of the 7 variables in the table increase; while the variability in the rates of change of 4 out of 6 for the autonomic effect *decrease* (though for high frequency, the change is not statistically significant).

### Frailty index and energetic efficiency

**Fig. 10:**
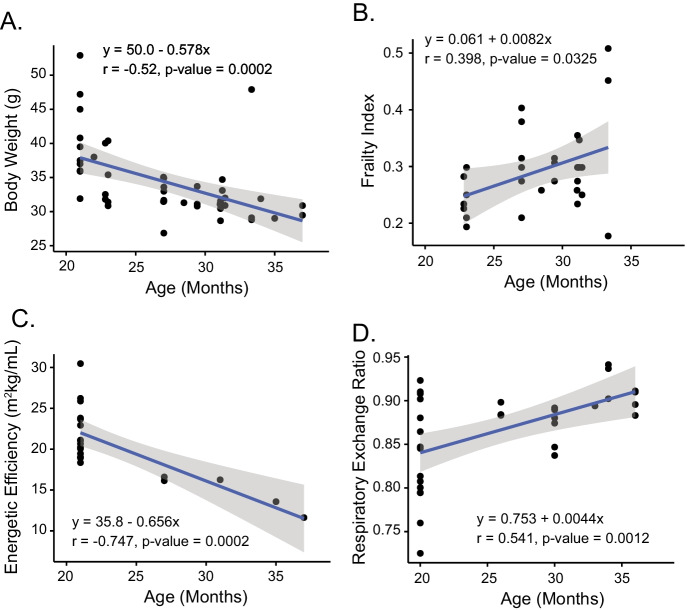
Correlations between age and **A** body weight, **B** frailty index, **C** energetic efficiency, and **D** respiratory exchange ratio in advanced age measured in a cross-sectional C57/BL6 mouse cohort

We assessed whole organism frailty to determine whether the deterioration of the intrinsic SAN RR interval variability signatures that underlie the marked increase in the mean intrinsic RR interval observed in mice of advanced age in our LL longitudinal cohort is paralleled by an increase in organ-wide whole-body frailty in a separate cross-sectional subset of C57/BL6 mice of advanced age (Fig. 10). Frailty scores in these mice increased progressively beyond 21 months of age (Fig. 10A) and were paralleled by a body weight loss, similar to the body weight loss observed in our LL cohort (Fig. 1B).

We next examined the relationship of the longitudinal *R*Δ of body weight change to the *R*Δ of mean RR interval change of LL mice. How the *R*Δ of body weight changed over the life course for LL mice is shown in Fig. S7A (replotted from Fig. 1B), and the rate at which basal and intrinsic mean RR changes over the life course is shown in Fig. S7B. Note that the body weight began to decline in individual mice (*R*Δ becomes negative between 18 and 21 months of age and becomes negative beyond 21 months of age for all mice (Fig. S7A), and the mean basal and intrinsic RR intervals increased beyond 21 months of age (Fig. S7B). At 24 months of age, the *R*Δ of body weight was correlated with the *R*Δ of both the basal and intrinsic mean RR (Fig. S7C) interval changed with age and the *R*Δ of the autonomic signature (Fig. S7D).

It is well known that frailty associated with loss of body weight is accompanied by a reduction in the efficiency of oxygen utilization [40, 41], which can be quantified as energetic efficiency measured as oxygen consumed during walking/speed of kinetic work performed [26]. Energetic efficiency measured cross-sectionally also became reduced with advanced age (Fig. 10C) and was associated with an increase in the respiratory exchange ratio accompanied by a reduction in energy efficiency (Fig. 10D).

These indices included an increase in a noncardiac, constitutional, whole-body frailty index, decreased energetic efficiency, and increased respiratory exchange ratio.

## Discussion

We conducted a unique longitudinal study of mouse RR interval variability and mean RR interval analyses in EKG time series recorded at 3-month intervals from 6 months to the end of life (approximately 30 months). By measuring RR interval variability prior to and during double autonomic blockade in a subset of mice that achieved the median cohort life span (24 months) or survived beyond this age, we were able to assess how both the intrinsic SAN and autonomic signatures within EKG RR interval time series changed over the entire life course, particularly in advanced age. We assessed mouse-specific rates of change (*R*Δ) of SAN and autonomic signatures throughout the adult mouse life span and how these signatures vary within the same mouse over time to determine the rates at which the mean intrinsic RR interval increases during late life. The emergence and progression of alterations in SAN signatures within EKG RR interval time series cause the mean RR interval to increase over this age range progressively. Increased net sympathetic autonomic modulation of this frail SAN masks, to a large extent, the signs of SAN frailty within the RR interval time series that emerge during late life.

### The relationship of SAN frailty to body-wide frailty

In an additional cross-sectional C57/BL6 mouse cohort, we showed that beyond about 21 months of age in which marked disorder of SAN function emerged, a body-wide frailty index [13], not inclusive of cardiac parameters, also sharply increased (Fig. 10B). An increased frailty score with advanced age has also been demonstrated using this index in prior cross-sectional studies in mice [14, 17, 26]. A prominent element within this frailty index is body weight loss, which not only occurred in our cross-sectional cohort, but also began to occur in all mice in our LL longitudinal cohort at 18 months of age (Fig. 10). The change in body weight in our LL cohorts was directly correlated with the ROC of the autonomic effect on intrinsic mean RR and part of the set of variables in which the ROC of mean intrinsic RR interval variability parameters clustered with the ROC of mean intrinsic RR intervals. The increase in the frailty index and loss of body weight during late life in our cross-sectional cohort was accompanied by a loss of energy efficiency (Fig. 10C) and an increase in the respiratory exchange ratio (Fig. 10D). The reduced energy efficiency is likely a direct contributor to the declining body weight in these animals. As reduced energy efficiency with age is generally associated with impaired mitochondrial function and increased production of reactive oxygen species, this may also promote both the cardiac and noncardiac frailty that we observe. A longitudinal assessment of body weight, walking speed, strength, endurance, and physical activity as markers of frailty observed that frailty onset at 17 months of age and increased during aging [42].

In the presence of these other noncardiac frailty markers in mice of advanced age, we may interpret the marked changes in increased intrinsic SAN RR variability that underlies the marked increase in the intrinsic mean RR interval that emerges in late life to be a signature of progressive SAN frailty.

### The signatures of SAN frailty in long-lived mice of advanced age

Most measured RR interval variability parameters and mean RR interval remained stable up to about 18 months, but beyond 21 months of age changed dramatically. Between 21 and 30 months, most *intrinsic* RR variability parameters (measured during dual autonomic blockade) manifested progressive changes, reflecting dysfunction of mechanisms *intrinsic* to pacemaker cells residing within the SAN [5], causing the mean intrinsic RR interval to increase with advanced age. Signatures of functional deterioration that occur within and among cells comprising SAN tissue underlying the marked increase in the mean intrinsic RR interval beyond 21 months of age are broadcast to the body surface as “heart beat music” [43] and can be heard on numerous EKG RR interval variability “channels,” including those that broadcast signals in the time, frequency, nonlinear, and fragmentation domains. The time at which successive RR intervals occur within a time series requires *memory* within SAN pacemaker cells created during prior beats [5]. Because each heartbeat is an emergent phenomenon [9], i.e., the SAN is not a metronome with a pre-written score.

Specific components of this SAN frailty signature include the following: (1) increased nonlinear intrinsic RR interval variability within short- and long-range correlations of RR intervals; (2) reductions in the %HF power within intrinsic RR interval rhythms; (3) increase in %VLF intrinsic power within RR rhythms; (4) increased long-range detrended RR interval fluctuations; (5) progressive increase in the percentage of intrinsic RR interval segments exhibiting alternans. In toto, these RR interval variability signatures of advanced age reflect an inability of SAN tissue to generate high-frequency (i.e., short) RR intervals, a process that requires memory of prior RR intervals, which requires synchronization among molecular functions, suggesting that in advanced age, the kinetics of molecular functions become reduced, leading to desynchronization of these functions within and among SAN pacemaker cells. Cluster analyses of mouse-specific rates of change (*R*Δ) for the intrinsic RR interval variability correlations among longitudinal *R*Δ of the clustered parameters enabled the identification of intrinsic SAN RR interval variability signature components that underlie the marked increase in the mean intrinsic RR interval emerged beyond 21 months of age in LL mice (Table S2, Fig. 7).

Increases of intrinsic SD1 and SD2 mean that RR interval variability within both short-range and long-range correlations of RR intervals generated within and among populations of SAN pacemaker cells increases in advanced age in LL mice as SAN frailty begins to emerge (Fig. 3). The nonlinearity of RR interval variability within short- and long-range RR interval correlations also increases with aging, as reflected in shifts in intrinsic SD1:SD2 over the late-life course. A youthful profile of SD1, SD2, and SD1:SD2 likely requires high-frequency signal processing because the rate at which intrinsic %HF power is lost between 21 and 30 months correlates with the rate at which intrinsic SD1:SD2 changes (Fig. 3). Significant increases in SD1, SD2, and correlations between the rates at which intrinsic SD1:SD2 and %HF power components and decline in a given mouse are associated with the rate at which mean intrinsic RR interval increases in that mouse would appear to reflect a loss of memory concerning when to generate the next AP as SAN frailty emerges because high frequency and nonlinearity of signals emerging from within and among populations of SAN cells inform on the memory that is required to maintain short (high frequency) and fairly regular (synchronized) RR intervals. The strong positive correlations of the rates at which HF components of RR intervals within the time series decreased between 21 and 30 months in each mouse and the rates at which SD1:SD2 decreased in that mouse suggest that reductions in the kinetics of pacemaker cell clock functions and desynchronization among these functions [5] may be linked to increased nonlinearity of RR interval variability within both the short- (SD1) and long-term (SD2) RR interval correlations buried within the EKG RR interval time series. Rates at which intrinsic %HF power is lost and nonlinearity in (SD1:SD2) increase in each mouse during advanced age were correlated with the rate at which mean intrinsic RR interval increased in that mouse, suggesting that this prolongation of the mean RR interval reflects reduced ability of pacemaker cells in the frail SAN to process high-frequency signals.

The tight coupling between the rate at which the reduction in intrinsic %HF power and the rate at which intrinsic %VLF power increased, in the absence of a change in the rate of total power (Figs. 5 and 8), also points to a reduction in the kinetics of intracellular Ca^2+^ and membrane potential transitions, leading to variability in mechanisms within and among SAN cells that relate to SAN pacemaker cell memory loss during the emergence of SAN frailty [5, 44, 45]. A reduction in these kinetics leads to desynchronization of molecular actions within SAN pacemaker cells associated with a loss of memory of synchronization effects created by the prior action potential [5].

The rates at which intrinsic DFA, slope coefficient *α*_2_, another measure of long-term RR interval variability measure, and intrinsic %VLF power increased beyond 21 months of age in a given mouse were also significantly correlated with the rates at which the mean RR interval increases. Directionally opposite changes in the rate at which intrinsic *α*_2_ (increased) and SD1:SD2 decreased during advanced age likely mean that the rate at which *α*_2_ increases reflects the rate at which HF signal processing is lost within and among populations of SAN cells. Thus, similar to the rate at which %VLF power increases beyond 21 months of age, the rate at which *α*_2_ increases is also correlated with the rate at which the mean intrinsic RR interval increases. The strong positive correlations between the rates at which %VLF power and *α*_2_ increased in a given mouse between 21 and 30 months of age indicate that the rate at which *α*_2_ increases reflects an increase in RR variability of VLF events that occur within long-range RR interval (*α*_2_) correlations.

Increased SD1, SD2, SD1:SD2, increases in the DFA and slope coefficient *α*_2_, and shifts from intrinsic %HF power to intrinsic %VLF power underlie the increase in *intrinsic* percent alternating segments (PAS) within the EKG time series of LL mice of advanced age that characterize SAN frailty beyond 21 months of age. This increase in the percentage of alternating long-short segments within the EKG interval time series in LL mice of advanced age (i.e., increase in PAS) is reminiscent of action potential and calcium alternans that characterize dysfunctions within and among SAN pacemaker cells that underlie cardiac arrhythmias [46]. In short, the most characteristic signature of SAN frailty is a loss in the ability of the SAN pacemaker cells to generate HF signals, resulting in reduced synchronization among the ensemble of cells. This translates into a markedly prolonged interval at which a given heartbeat follows the previous beat, resulting in a marked increase in the mean RR interval or reduction in mean intrinsic HR.

RR interval variability of the aforementioned mechanisms and the mean RR interval in the *basal* state result from modulation of the mechanisms intrinsic to SAN pacemaker cell that determine the *intrinsic* RR interval variability. The net result of these autonomic signatures on the intrinsic RR interval signatures was to reduce the basal RR interval to a more youthful level. Further, because the RR interval became reduced (i.e., HR was increased), this autonomic signature was net sympathetic in nature. Thus, age-associated changes in intrinsic RR interval signatures (heartbeat music broadcast to the body surface) reflected in the EKG time series are the net result of multiple changes within and among SAN cells and their responsiveness to autonomic input. Nevertheless, even after this autonomic input, the basal state mean RR interval within a given mouse was not fully restored to its value at younger ages because the autonomic signatures could not fully restore the RR interval variability signatures to their youthful levels.

### Cell and molecular bases of RR interval variability signatures and mean RR interval

#### How do pacemaker cells keep time?

Both experimental and theoretical perspectives have led to the idea that a coupled-oscillator system intrinsic to individual pacemaker cells drives normal, youthful automaticity [2]. Briefly, the sarcoplasmic reticulum (SR), a Ca^2+^ oscillator (“calcium clock”) within SAN pacemaker cells, couples to an ensemble of current oscillators located on the cell surface membrane (“membrane clock”) that includes Na^+^/Ca^2+^ exchanger proteins, Ca^2+^, K^+^, Na^+^ ion channels, and Na^+^/K^+^ ATPase proteins. The timekeeping by the Ca^2+^ clock and its interaction with surface membrane electrogenic molecules create a coupled-clock system that regulates the spontaneous pacemaker cells’ AP firing rate, i.e., normal automaticity [2]. Constitutive activation of a non-G protein coupled, Ca^2+^ calmodulin-dependent adenyl cyclase (type 8), is the biochemical driver of the coupled-clock system. The clocks mutually entrain each other in a feed-forward manner on a beat-to-beat basis generating recurrent rhythmic electrochemical signals that underlie recurrent action potential (AP) cycles. [2]. Modulation of the effectiveness or fidelity of clock coupling, determined by synchronization of kinetics of activation/inactivation mechanisms of clock molecules that regulate Ca^2+^ and membrane potential, controls the rhythm and rate of AP firing.

Ca^2+^ is the oscillatory substrate of the SR clock, which spontaneously cycles (pumps into itself and spontaneously releases Ca^2+^ in a roughly periodic manner) [44]. SR-generated local calcium releases (LCRs) occur during diastole to promote the spontaneous diastolic surface membrane potential depolarization that culminates in the firing of the subsequent AP. The speed at which the SR pumps and cycles Ca^2+^ locally within SAN pacemaker cells to generate LCRs is a critical factor in determining the extent of clock coupling, which controls the rate at which the pacemaker cells fire spontaneous APs. The time following a previous beat at which the next AP will occur is regulated by memory and encoded by the Ca^2+^ charge on the SR (SR Ca^2+^ load), which controls the timing of LCRs from the SR. Encoding of short-term memory (SR Ca^2+^ charge) is influenced by the following: (1) the recent past history of AP firing intervals, which modulates the cell and SR Ca^2+^ load on a beat-to-beat basis, and (2) the extent to which clock molecules are phosphorylated, which also encodes longer-term memory within the coupled-clock pacemaker cell system.

Phosphorylation of PLB and RyR, proteins of the SR, and of the SR itself resulting from constitutive AC activation, i.e., in the absence of β-AR stimulation, results in downstream high (relative to ventricular myocytes) basal levels of PKA-dependent protein phosphorylation of these, and other cellular proteins involved in SAN pacemaker cell Ca^2+^ homeostasis. Basal PDE and phosphoprotein phosphatase activities limit cAMP and cAMP-dependent phosphorylation levels to maintain the set point of Ca^2+^-AC-cAMP-PKA-Ca^2+^ signaling near the midpoint of its basal state range [2]. The extent to which clock proteins are phosphorylated determines the synchronization of their kinetic functions within each clock, determining the extent to which the calcium and membrane clocks are coupled.

Autonomic input to SAN pacemaker cells modulates the same intrinsic clock functions that drive AP firing in the absence of autonomic receptor stimulation by increasing or reducing the duration and variability of inter-AP firing intervals due to a direct or indirect impact on memory encoded within the SR (the SR calcium load), thus shifting the mean AP firing interval to higher or lower values. Adrenergic receptor activation increases cell Ca^2+^ levels and clock protein phosphorylation, thereby increasing the fidelity of entrainment of clock coupling [5]. As the effectiveness of clock coupling increases in response to adrenergic input, AP interval variability and mean AP firing interval become reduced, and thus, the mean AP firing interval becomes reduced [31, 47]. Conversely, reduced effectiveness of clock coupling in response to parasympathetic autonomic input reduces clock coupling leading to increases in AP firing interval variability and to prolongation of the mean AP firing interval [5].

#### Cellular and molecular bases of RR interval variability signatures of the frail SAN

Both age-associated reductions in clock molecule expression and reduced synchronization of molecular activation within individual pacemaker cells are root causes of SAN “frailty” in advanced age [45, 48]. A reduction in mean basal AP firing rate in aged SAN pacemaker cells occurs in the context of sluggish Ca^2+^ cycling: a reduced Ca^2+^ influx rate in response to an AP and a reduced rate of Ca^2+^ removal from the cytosol both contribute to compromised SR Ca^2+^ cycling. Studies in permeabilized SAN pacemaker cells that permit direct assessment of spontaneous SR Ca^2+^ cycling in a fixed physiological [Ca^2+^] milieu in the absence of an AP impact on the SR Ca^2+^ load indicate that the SR calcium load (a key determinant of encoding near-term memory of the coupled-clock system) is reduced in pacemaker cells isolated from 24-month-old mice compared to 8-month mice. In SAN cells with intact sarcolemma function (that fire spontaneous APs), the LCR size, number, and duration are reduced in SAN cells isolated from 24- vs. 8-month mice, and LCRs occur at a later time following the prior AP in cells from older mice. Furthermore, the effect of phosphodiesterase (PDE) inhibition to promote phosphorylation of clock proteins and synchronization of their functions and to increase memory encoding within the SR (i.e., to increase the SR Ca^2+^ load) are reduced in SAN cells from aged vs. young adult mice [45]. These age-associated changes in SR Ca^2+^ cycling coincide with reduced expression of crucial SR Ca^2+^ cycling proteins, including SR Ca^2+^ pump (Ca^2+^-ATPase or SRCA2) ryanodine receptors and Na^+^/Ca^2+^ exchanger in aged vs. younger adult SAN cells. Further, the phospholamban (PLB) ratio to SERCA2 increases in advanced age, suggesting that the inhibition of SERCA by PLB could be higher in advanced age [49]. Thus, a reduction in Ca^2+^ cycling kinetics in aged SAN cells appears to be involved in the age-associated reduction in the basal intrinsic AP firing rate and reduced response to a cyclic AMP PDE inhibition.

In addition, important age-associated changes in the mRNA profiles of different ion channel and exchanger genes have recently been described in the atrium and SAN [50]. The L-type and I_f_ in mouse SAN cells decrease in advanced age [50]. There is an age-associated decrease in SAN RyR, KV1.5, and HCN1 gene expression in rat and an increase in Nav1.5, Navβ1, and Cav1.2 gene expression [50–53]. Age-associated changes to pharmacological inhibition of functions of other clock molecules have been documented: SAN pacemaker cells from aged rats (24 month) vs. 3-month-old rats are more sensitive to blockade of neuronal Na^+^ channel by TTX or to blockade of funny current (I_f_) by Cs^2+^ or ivabradine [53]. Pacemaker cells from aged guinea pig are more sensitive to blockade of L-type blocker by nifedipine [54].

But normal SAN function goes well beyond individual pacemaker cells because rhythmic impulses that emerge from the SAN result from the synchronization of heterogeneous, subcellular Ca^2+^ signals within and among SAN tissue-resident pacemaker cells [9]. Thus, the initiation of each heartbeat within the SAN is an emergent phenomenon, and in addition to age-associated deficits within individual SAN cells, the ability to synchronize heterogeneous local signals among cells may be compromised in advanced age. Further, age-associated cell matrix remodeling, including fibrosis [53], compounds the disordered state within the frail SAN. The leading pacemaker site does not appear to be affected by age in rabbits. The number of cells in aged human (50 vs. 75 years) SAN tissue decreases [55]. SAN Cx43 protein expression decreases with age in rat; however, the expression of other cardiac connexins, Cx40 and Cx45, does not differ with age [38]. As noted, sick sinus syndrome is associated with abnormal impulse initiation and propagation from the SAN and occurs most commonly in older patients [56]. In mice exhibiting sick sinus syndrome, there is an increase in pacemaker cell apoptosis, a reduction in the number of cells, and CaMKII activity is increased [57]. The corrected SAN recover time increased by 43% between 3 and 25 months of age in rats [58].

The sensitivity of the beating rate responses of the intact, isolated SAN to both muscarinic and adrenergic receptor activation becomes decreased in advanced age [38, 59]. Although the sensitivity to autonomic neurotransmitters becomes reduced with advancing age [59–61], an age-associated shift in sympathetic and vagal nerve impulses in vivo partially preserves the resting HR. In older humans, perturbations from the basal state elicit a reduced increase in HR and are associated with a greater neurotransmitter and epinephrine hormone spill over into the circulation [62].

Additional perspectives on how cardiac pacemaker activity and its neural control changed with age are reviewed in [15, 63]. Ultimately a deterioration in SAN pacemaker function, due in large measure to a reduction in the kinetics of coupled-clock functions that drive SR Ca^2+^ cycling and its response to a cAMP-dependent pathway activation, underlies the age-associated reduction in the intrinsic SAN AP firing rate at rest and in the reduction in the acceleration of heart rate during exercise.

In the present study, the age-associated reduction in the kinetics of Ca^2+^ cycling and the reduction in cAMP-PKA-dependent stimulation of Ca^2+^ cycling observed in mouse SAN are strikingly similar observations compared with how aging affects rat ventricular myocytes. Specifically, in ventricular myocytes, Ca^2+^ removal from the cytosol following AP Ca^2+^ release is slowed [64], due in part to a reduced expression of SERCA2 [39, 65], and this results in a prolonged contraction [39, 64]. The increase in SR protein phosphorylation in response to β-AR stimulation of ventricular cells becomes reduced with advancing age [66], and this is accompanied by a reduction in the β-AR-induced augmentation of the AP-induced SR Ca^2+^ release, acceleration of Ca^2+^ removal from the cytosol, and reduced augmentation of contraction amplitude and relaxation [61, 67]. A coordinated diminution in the function of these molecules in SAN and ventricular myocytes with aging would confer a protective adaptation: due to slower Ca^2+^ cycling in aged vs. adult ventricular cells in response to accelerated electrical impulses, aged ventricular myocytes become Ca^2+^ overloaded [64] and generate spontaneous abnormal SR-generated Ca^2+^ that can trigger abnormal APs [68]. Slower SR Ca^2+^ cycling in SAN pacemaker cells in response to β-AR stimulation results in reduced APs emanating from the SAN and conducted to ventricular cells. The reduction of the increase in heart rate during stress (e.g., β-AR stimulation during exercise) protects ventricular cells in the aged heart from Ca^2+^ overload and arrhythmias. Other molecules of the coupled pacemaker clock system, e.g., PKA signaling and mitochondrial ATP production proteins, also may change with age and require further study.

## Supplementary Information

Below is the link to the electronic supplementary material.

Supplementary file1 (PDF 4229 KB)
