# Supplementary material for: Emergence of heartbeat frailty in advanced age I: perspectives from life-long EKG recordings in adult mice
Source: GeroScience. 2022 Jun 27;44(6):2801–30. doi: 10.1007/s11357-022-00605-4 (PMC9768068; doi:10.1007/s11357-022-00605-4)
Supplement: Supplementary file 1 — Supplementary file1 (PDF 4229 KB) [file 11357_2022_605_MOESM1_ESM.pdf]

Emergence of Heartbeat Frailty in Advanced Age I: Perspectives from Life-Long EKG Recordings in Adult Mice

by

Jack M Moen, Christopher H. Morrell, Michael G. Matt, Ismayil Ahmet, Syevda Tagirova, Moran Davoodi, Michael Petr, Shaquille Charles, Rafael de Cabo, Yael Yaniv, and Edward G Lakatta

Supplement

Supplement Table S1A. Descriptive statistics of HR and HRV parameters in Long Lived mice.

		Intrinsic State (during double autonomic blockade)								
	Age	6	9	12	15	18	21	24	27	30
**Time Domain**										
Mean RR (ms)	n*	30	28	29	30	28	29	28	16	3
	mean	135.86	133.81	142.93	131.73	139.33	138.09	151.32	196.1	154.75
	sd	7.5543	6.9557	6.3442	6.7944	10.108	9.7206	9.8015	14.478	69.227
SD RR (ms)	n	30	28	29	30	28	29	28	16	3
	mean	1.91	1.616	1.8154	1.5356	2.1781	1.802	2.2878	5.1416	29.159
	sd	0.5472	0.5025	0.604	0.6912	2.306	0.8568	1.3248	3.585	40.273
**Frequency Domain**										
Total Power PSD (ms^2)	n	30	28	29	30	28	29	28	16	3
	mean	1.7269	1.2817	1.6601	1.3501	10.286	2.2217	5.43	19.707	44.669
	sd	1.0202	0.7197	1.0052	1.6521	34.37	2.6958	12.486	23.287	37.742
VLF (%)	n	30	28	29	30	28	29	28	16	3
	mean	8.5982	10.944	14.587	17.682	15.438	15.317	18.881	29.891	40.329
	sd	4.6037	8.8516	13.031	12.701	10.15	10.48	10.178	15.225	30.462
LF (%)	n	30	28	29	30	28	29	28	16	3
	mean	8.9183	10.891	12.756	11.72	14.401	16.543	13.281	21.088	65.082
	sd	5.8251	6.2324	9.6027	7.3111	12.266	10.575	11.947	13.919	31.832
HF (%)	n	30	28	29	30	28	29	28	16	3
	mean	77.513	72.832	65.299	62.606	59.341	53.457	48.169	26.461	47.794
	sd	10.752	15.446	20.821	20.871	22.597	22.212	22.361	20.61	45.237
LF_TO_HF Ratio	n	30	28	29	30	28	29	28	16	3
	mean	0.1327	0.1759	0.2616	0.2495	0.2704	0.3572	0.5631	2.1338	27.815
	sd	0.1024	0.1239	0.2192	0.179	0.2061	0.2518	1.2613	4.2574	41.361
β (N.U.)	n	30	28	29	30	28	29	28	16	3
	mean	-1.383	-1.315	-1.233	-1.371	-1.325	-1.231	-1.289	-1.282	24.405
	sd	0.22	0.2014	0.3093	0.2907	0.3397	0.3892	0.3999	0.5426	44.214
**Non-linear Domain**										
SD1 (ms)	n	30	28	29	30	28	29	28	16	3
	mean	2.0242	1.7053	1.8506	1.4973	2.1036	1.6488	2.0917	3.7887	27.904
	sd	0.6206	0.5874	0.7486	0.8172	2.4967	0.8243	1.4499	3.3762	41.252
SD2 (ms)	n	30	28	29	30	28	29	28	16	3
	mean	1.769	1.5027	1.7363	1.5254	2.0875	1.861	2.3579	5.7731	30.013
	sd	0.4923	0.4351	0.5198	0.6275	1.9782	0.9276	1.2871	3.9549	39.644
SD Ratio	n	30	28	29	30	28	29	28	16	3
	mean	1.1393	1.114	1.0434	0.9656	0.9819	0.8964	0.8476	0.6729	0.7431
	sd	0.1411	0.2005	0.2751	0.2686	0.2441	0.2751	0.2726	0.3452	0.2346
α_1 (N.U.)	n	30	28	29	30	28	29	28	16	3
	mean	0.267	0.3123	0.3605	0.3713	0.3633	0.4176	0.3727	0.531	25.642
	sd	0.1011	0.1207	0.166	0.1412	0.1132	0.1439	0.1956	0.3097	43.143
α_2 (N.U.)	n	30	28	29	30	28	29	28	16	3
	mean	0.5837	0.6022	0.6663	0.6865	0.6718	0.6429	0.7434	0.8097	25.578
	sd	0.1465	0.1759	0.203	0.2273	0.1965	0.1916	0.1883	0.2572	43.198
Sample Entropy (N.U.)	n	30	28	29	30	28	29	28	16	3
	mean	1.3289	1.3638	1.3767	1.4454	1.447	1.4457	1.332	1.0989	25.79
	sd	0.2012	0.2971	0.2781	0.3429	0.3453	0.2827	0.3164	0.4287	43.016

**Heart Rate Fragmentation**										
PIP (%)	n	30	28	29	30	28	29	28	16	3
	mean	56.331	59.513	61.148	59.923	60.791	59.672	59.179	59.168	60.231
	sd	4.8508	5.1295	4.6558	4.3934	4.9786	4.3829	5.2608	5.1327	17.504
IALS (N.U.)	n	30	28	29	30	28	29	28	16	3
	mean	0.5645	0.5963	0.6128	0.6004	0.6092	0.598	0.5931	0.5935	25.505
	sd	0.0486	0.0513	0.0466	0.044	0.0499	0.0439	0.0527	0.0514	43.261
PSS (%)	n	30	28	29	30	28	29	28	16	3
	mean	66.12	73.606	75.775	74.967	74.546	69.684	62.799	63.905	60.173
	sd	11.908	13.117	10.695	9.0841	10.007	11.084	15.702	7.9964	21.741
PAS (%)	n	30	28	29	30	28	29	28	16	3
	mean	10.326	12.559	13.706	11.966	14.555	14.479	20.067	27.342	40.851
	sd	7.1928	10.623	11.989	9.0871	11.381	9.5201	10.646	7.7683	31.79

Supplement Table S1B. Descriptive statistics of HR and HRV parameters in Long Lived mice.

		Basal State								
	Age	6	9	12	15	18	21	24	27	30
**Time Domain**										
	n*	30	28	29	30	28	29	28	17	3
Mean RR (ms)	mean	135.8	135.7	140.8	136.3	136.2	138.4	139.6	151.2	147.3
	sd	8.234	9.498	10.56	11.23	13.63	12.74	14.19	15.59	22.16
	n	30	28	29	30	28	29	28	17	3
SD RR (ms)	mean	2.876	3.146	2.895	2.816	2.83	3.159	3.697	4.504	4.826
	sd	1.128	2.352	1.186	1.523	1.777	2.009	2.86	3.176	5.607
**Frequency Domain**										
	n	30	28	29	30	28	29	28	17	3
Total Power PSD	mean	4.224	6.77	4.571	4.578	5.15	6.646	11.09	15.46	29.69
(ms^2)	sd	3.053	14.23	3.708	5.816	9.062	9.796	20.68	19.38	48.39
	n	30	28	29	30	28	29	28	17	3
VLF (%)	mean	28.06	33.73	33.41	35.48	36.04	33.74	36.29	26.1	25.86
	sd	11.38	13.6	14.43	14.2	17.82	16.9	17.74	15.79	1.46
	n	30	28	29	30	28	29	28	17	3
LF (%)	mean	8.607	7.895	10.63	7.737	8.464	13.53	11.7	26.54	51.58
	sd	7.211	4.228	9.871	5.082	7.682	12.78	10.15	15.2	18.1
	n	30	28	29	30	28	29	28	17	3
HF (%)	mean	47.77	41.38	41.24	35.73	37.9	34.9	34.32	42.16	18.32
	sd	16.12	17.7	22.16	20.53	21.96	19.66	23.73	27.57	8.872
	n	30	28	29	30	28	29	28	17	3
LF_TO_HF Ratio	mean	0.207	0.329	0.427	0.561	0.345	0.502	0.509	1.41	3.731
	sd	0.22	0.525	0.654	1.483	0.366	0.543	0.534	1.651	3.04
	n	30	28	29	30	28	29	28	17	3
β (N.U.)	mean	-1.64	-1.66	-1.5	-1.71	-1.67	-1.46	-1.35	-1.06	-0.97
	sd	0.197	0.234	0.353	0.168	0.251	0.504	0.529	0.463	0.722
**Non-linear Domain**										
	n	30	28	29	30	28	29	28	17	3
SD1 (ms)	mean	2.261	2.18	2.218	2.014	2.069	2.063	2.738	3.591	2.493
	sd	0.964	1.148	1.204	1.56	1.694	1.176	3.013	3.033	2.646
	n	30	28	29	30	28	29	28	17	3
SD2 (ms)	mean	3.317	3.781	3.324	3.309	3.32	3.827	4.238	4.892	6.338
	sd	1.364	3.225	1.372	1.709	2.024	2.664	2.959	3.723	7.468
	n	30	28	29	30	28	29	28	17	3
SD Ratio	mean	0.703	0.649	0.68	0.596	0.632	0.591	0.597	0.81	0.442
	sd	0.195	0.229	0.291	0.265	0.276	0.254	0.331	0.412	0.064
	n	30	28	29	30	28	29	28	17	3
α_1 (N.U.)	mean	0.346	0.394	0.443	0.427	0.4	0.501	0.496	0.599	0.975
	sd	0.147	0.224	0.235	0.245	0.205	0.206	0.247	0.3	0.285
	n	30	28	29	30	28	29	28	17	3
α_2 (N.U.)	mean	1.008	1.097	1.041	1.096	1.088	1.035	1.081	0.742	0.616
	sd	0.203	0.13	0.2	0.216	0.257	0.268	0.271	0.255	0.155
	n	30	28	29	30	28	29	28	17	3
Sample Entropy	mean	1.468	1.449	1.436	1.389	1.412	1.402	1.34	1.328	1.353
(N.U.)	sd	0.301	0.372	0.287	0.33	0.31	0.319	0.339	0.399	0.511

**Heart Rate Fragmentation**										
	n	30	28	29	30	28	29	28	17	3
PIP (%)	mean	56.14	58.85	60.29	61.08	60.49	60.08	56.8	56.9	45.24
	sd	4.899	4.479	3.975	4.816	5.397	4.215	5.473	7.813	3.703
	n	30	28	29	30	28	29	28	17	3
IALS (N.U.)	mean	0.563	0.59	0.604	0.612	0.606	0.602	0.569	0.57	0.454
	sd	0.049	0.045	0.04	0.048	0.054	0.042	0.055	0.078	0.037
	n	30	28	29	30	28	29	28	17	3
PSS (%)	mean	74.53	79.53	81.49	79.9	78.64	76.43	67.73	61.76	42.35
	sd	9.979	10.26	10.3	12.43	11.7	10.68	12.42	12.81	5.667
	n	30	28	29	30	28	29	28	17	3
PAS (%)	mean	11.16	10.86	10.29	14.83	14.18	12.95	15.9	23.88	10.42
	sd	9.803	9.033	8.032	10.37	13.53	7.534	11.49	12.48	5.318

Supplement Table S1C. Descriptive statistics of HR and HRV parameters in Long Lived mice.

	Basal - Intrintic = Effect of Autonomic Input								
Age	6	9	12	15	18	21	24	27	30
**Time Domain**									
	30	28	29	30	28	29	28	16	3
Mean RR (ms)	-0.04	1.85	-2.18	4.57	-3.13	0.27	-11.70	-46.52	-7.42
	5.72	8.34	7.80	8.57	11.40	7.68	8.26	8.10	85.35
	30	28	29	30	28	29	28	16	3
SD RR (ms)	0.97	1.53	1.08	1.28	0.65	1.36	1.41	-0.72	-24.33
	1.04	2.18	0.94	1.09	1.64	1.58	2.22	4.39	34.89
**Frequency Domain**									
	30	28	29	30	28	29	28	16	3
Total Power PSD	2.50	5.49	2.91	3.23	-5.14	4.42	5.66	-4.91	-14.98
(ms^2)	2.84	14.07	3.27	4.58	33.22	8.54	10.83	30.19	35.61
	30	28	29	30	28	29	28	16	3
VLF (%)	19.46	22.78	18.82	17.80	20.60	18.43	17.41	-4.44	-14.47
	12.53	13.50	11.68	13.69	16.01	14.56	15.47	16.63	31.76
	30	28	29	30	28	29	28	16	3
LF (%)	-0.31	-3.00	-2.12	-3.98	-5.94	-3.01	-1.58	6.21	-13.50
	8.83	5.53	8.89	6.16	10.76	8.84	8.14	22.54	25.71
	30	28	29	30	28	29	28	16	3
HF (%)	-29.74	-31.46	-24.06	-26.88	-21.44	-18.56	-13.85	18.07	-29.47
	20.64	17.23	20.16	22.50	26.98	26.14	28.10	22.61	53.10
	30	28	29	30	28	29	28	16	3
LF_TO_HF Ratio	0.07	0.15	0.17	0.31	0.07	0.14	-0.05	-1.05	-24.08
	0.24	0.49	0.59	1.38	0.34	0.47	1.16	4.56	38.54
	30	28	29	30	28	29	28	16	3
β (N.U.)	-0.261	-0.341	-0.268	-0.336	-0.348	-0.228	-0.058	0.228	-25.378
	0.294	0.307	0.370	0.303	0.346	0.392	0.550	0.687	43.528
**Non-linear Domain**									
	30	28	29	30	28	29	28	16	3
SD1 (ms)	0.236	0.474	0.367	0.517	-0.034	0.414	0.646	-0.104	-25.411
	0.884	0.859	0.848	0.955	1.352	0.854	2.627	3.173	38.714
	30	28	29	30	28	29	28	16	3
SD2 (ms)	1.548	2.278	1.588	1.784	1.233	1.966	1.880	-1.073	-23.675
	1.284	3.085	1.182	1.365	1.792	2.181	2.172	5.404	32.533
	30	28	29	30	28	29	28	16	3
SD Ratio	-0.437	-0.465	-0.363	-0.370	-0.350	-0.306	-0.251	0.171	-0.301
	0.239	0.209	0.249	0.256	0.306	0.311	0.335	0.357	0.295
	30	28	29	30	28	29	28	16	3
α_1 (N.U.)	0.079	0.082	0.082	0.056	0.037	0.084	0.123	0.031	-24.667
	0.176	0.208	0.182	0.191	0.181	0.206	0.217	0.401	42.873
	30	28	29	30	28	29	28	16	3
α_2 (N.U.)	0.424	0.495	0.374	0.410	0.416	0.392	0.337	-0.062	-24.962
	0.226	0.203	0.169	0.282	0.290	0.238	0.262	0.248	43.302
	30	28	29	30	28	29	28	16	3
Sample Entropy	0.14	0.09	0.06	-0.06	-0.03	-0.04	-0.01	0.26	-24.44
(N.U.)	0.23	0.36	0.30	0.38	0.36	0.32	0.34	0.60	43.53

Heart Rate Fragmentation									
	30	28	29	30	28	29	28	16	3
PIP (%)	-0.19	-0.66	-0.86	1.15	-0.30	0.41	-2.38	-1.42	-14.99
	4.35	4.66	4.98	4.59	4.85	4.71	5.57	9.61	20.88
	30	28	29	30	28	29	28	16	3
IALS (N.U.)	-0.002	-0.007	-0.009	0.012	-0.003	0.004	-0.024	-0.015	-25.051
	0.044	0.047	0.050	0.046	0.049	0.047	0.056	0.096	43.275
	30	28	29	30	28	29	28	16	3
PSS (%)	8.41	5.92	5.71	4.93	4.10	6.75	4.93	-0.72	-17.82
	13.55	11.16	9.88	7.60	10.75	10.72	13.61	16.80	26.93
	30	28	29	30	28	29	28	16	3
PAS (%)	0.84	-1.70	-3.41	2.87	-0.37	-1.53	-4.17	-2.83	-30.43
	9.14	11.92	11.05	10.11	10.63	10.13	10.07	14.11	33.47

Table S3. Correlations and p-values of Mean RR with additional variables excluded from those that clustered with the increased mean RR interval in Fig. 6.

	Mean RR	SDRR	Total Power	LF	LF/HF	β	SD1	SD2	α_1	Sample Entropy	PIP	IALS	PSS
Mean RR : r		0.2485	0.0963	0.0926	0.0893	-0.0534	0.2391	0.2430	-0.0258	0.1376	0.3537	0.3648	0.2809
Mean RR : p		0.1854	0.6129	0.6266	0.6390	0.7794	0.2031	0.1958	0.8922	0.4684	0.0552	0.0475	0.1327
SDRR : r	0.2587		0.5317	0.0957	-0.0175	0.3991	0.9566	0.9692	-0.1033	-0.4317	0.0001	0.1899	0.1279
SDRR : p	0.1675		0.0025	0.6149	0.9269	0.0289	0.0000	0.0000	0.5869	0.0172	0.9997	0.3148	0.5006
Total Power : r	0.1554	0.9314		0.4895	0.3407	0.2988	0.4144	0.4752	0.2520	-0.1372	0.0347	0.0505	0.0091
Total Power : p	0.4122	8.2E-14		0.0060	0.0654	0.1087	0.0228	0.0080	0.1792	0.4698	0.8556	0.7911	0.9619
LF : r	-0.1159	0.3861	0.5291		0.8305	0.5644	-0.0094	0.1119	0.7343	-0.1685	-0.0540	-0.0202	-0.1941
LF : p	0.5421	0.0351	0.0026		1.4E-08	0.0012	0.9608	0.5559	3.9E-06	0.3734	0.7767	0.9154	0.3041
LF/HF : r	0.0393	0.4427	0.6258	0.7216		0.3529	-0.2171	0.1055	0.9259	-0.1505	0.0260	0.0954	-0.1583
LF/HF : p	0.8367	0.0143	0.0002	6.8E-06		0.0558	0.2492	0.5789	2.3E-13	0.4272	0.8917	0.6162	0.4035
β : r	0.0106	0.5640	0.6156	0.6493	0.5574		0.3978	0.3573	0.3180	-0.4953	-0.0080	0.1423	0.0889
β : p	0.9558	0.0012	0.0003	0.0001	0.0014		0.0295	0.0526	0.0868	0.0054	0.9667	0.4533	0.6404
SD1 : r	0.1446	0.8441	0.6910	0.2142	0.0355	0.4208		0.8685	-0.2847	-0.3777	0.0165	0.1527	0.1578
SD1 : p	0.4458	4.6E-09	2.4E-05	0.2558	0.8521	0.0206		5.0E-10	0.1273	0.0396	0.9310	0.4204	0.4051
SD2 : r	0.2798	0.9502	0.9531	0.4783	0.6192	0.5750	0.6451		0.0171	-0.4415	-0.0331	0.2089	0.0913
SD2 : p	0.1343	8.9E-16	4.4E-16	0.0075	0.0003	0.0009	0.0001		0.9285	0.0146	0.8620	0.2680	0.6313
α_1 : r	0.0177	0.4036	0.5433	0.5485	0.8007	0.4574	-0.0352	0.5920		-0.1303	0.0024	0.0481	-0.0751
α_1 : p	0.9260	0.0270	0.0019	0.0017	1.1E-07	0.0110	0.8537	0.0006		0.4927	0.9899	0.8006	0.6931
Sample Entropy : r	0.1937	-0.6270	-0.5650	-0.3840	-0.3243	-0.6726	-0.5670	-0.5778	-0.3180		-0.0095	-0.1335	-0.2226
Sample Entropy : p	0.3050	0.0002	0.0011	0.0362	0.0804	4.7E-05	0.0011	0.0008	0.0868		0.9604	0.4818	0.2371
PIP : r	0.1205	-0.2876	-0.3709	-0.5315	-0.4529	-0.3071	-0.1704	-0.3343	-0.3228	0.3053		0.8606	0.7326
PIP : p	0.5258	0.1233	0.0436	0.0025	0.0120	0.0988	0.3679	0.0710	0.0819	0.1009		1.1E-09	4.2E-06
IALS : r	0.1227	-0.2867	-0.3701	-0.5316	-0.4529	-0.3070	-0.1697	-0.3334	-0.3229	0.3055	1.0000		0.7631
IALS : p	0.5184	0.1245	0.0441	0.0025	0.0120	0.0989	0.3701	0.0718	0.0818	0.1006	0.0000		9.4E-07
PSS : r	-0.1424	0.3475	0.3579	0.3711	0.4174	0.3070	0.3041	0.3144	0.2964	-0.2977	-0.6038	-0.6043	
PSS : p	0.4530	0.0599	0.0521	0.0435	0.0217	0.0989	0.1023	0.0907	0.1117	0.1101	0.0004	0.0004	

The upper triangle contains the correlations in the early time period (before 21 months).
The lower triangle contains the correlations in the late time period (after 21 months).

Supplement Table S2. Statistical Analyses (*) of the effects of age on Basal, Intrinsic, and Autonomic Input on Intrinsic RR interval mean and RR interval variability in long lived mice.

	Intrinsic State		Basal State		Effect of Autonomic Input on Intrinsic	
Age (months)	6 to 21	21 to 30	6 to 21	21 to 30	6 to 21	21 to 30
Body Weight (g)			< 2.2e-16 ↑	3.5e-15 ↓		
**Time Domain**						
Mean RR (ms)	1.51E-06 ∽	< 2.2e-16 ↑		0.01172 ↑	0.0008 ∽	< 2.2e-16 ↑
SD RR (ms)		3.22e-6 ↑				0.0748 ↑
**Frequency Domain**						
Total Power PSD (ms^2)		0.0005 ↑		0.0984 ↑		
VLF (%)	0.0008 ↑	0.00577 ↑				1.31e-5 ↑
LF (%)	0.0021 ↑	0.0034 ↑	0.0385 ↑	1.04e-6 ↑		0.0576 ↓↑
HF (%)	7.33e-7 ↓	4.35e-5 ↓	0.0657 ↓↑			4.35e-05 ↓
LF TO HF Ratio	9.00e-6 ↑	0.0047 ↑		3.97e-6 ↑		
β (N.U.)			0.0071 ↑↓	0.05417 ↑		0.0316 ↓
**Non-linear Domain**						
SD1 (ms)		0.0044 ↑				
SD2 (ms)		5.39e-7 ↑				0.0317 ↑
SDRatio	3.95e-5 ↓	0.0253 ↓		0.0772 ∽		7.31e-5 ↓
α_1 (N.U.)	1.77e-5 ↑	0.0059 ↑	0.0355 ↑	0.0120 ↑		
α_2 (N.U.)		0.05697 ↑		7.29e-5 ↓		1.90e-7 ↑
Sample Entropy (N.U.)		0.00681 ↓				0.0793 ↓↑
**Heart Rate Fragmentation**						
PIP (%)	0.0002 ↑→		6.15e-5 ↑	0.0002 ↓		0.0444 ↑
IALS (N.U.)	0.0002 ↑→		6.22e-5 ↑	0.0002 ↓		0.0429↑
PSS (%)	0.0007 ↑↓	0.0318 ↓	0.0336 ↑↓	3.61e-6 ↓		0.0284 ↑
PAS (%)		0.00037 ↑		0.0053 ↑		

Notes:
* 1. p-values from repeated-measures mixed ANOVA models; 2. Effect of Autonomic Input on Intrinsic = Basal - Intrinsic in each mouse; 3. N.U. = no units. 4. ↑ variable is significantly increasing with age, ↓ variable is significantly decreasing with age, and ∽ the variable is significantly oscillating.

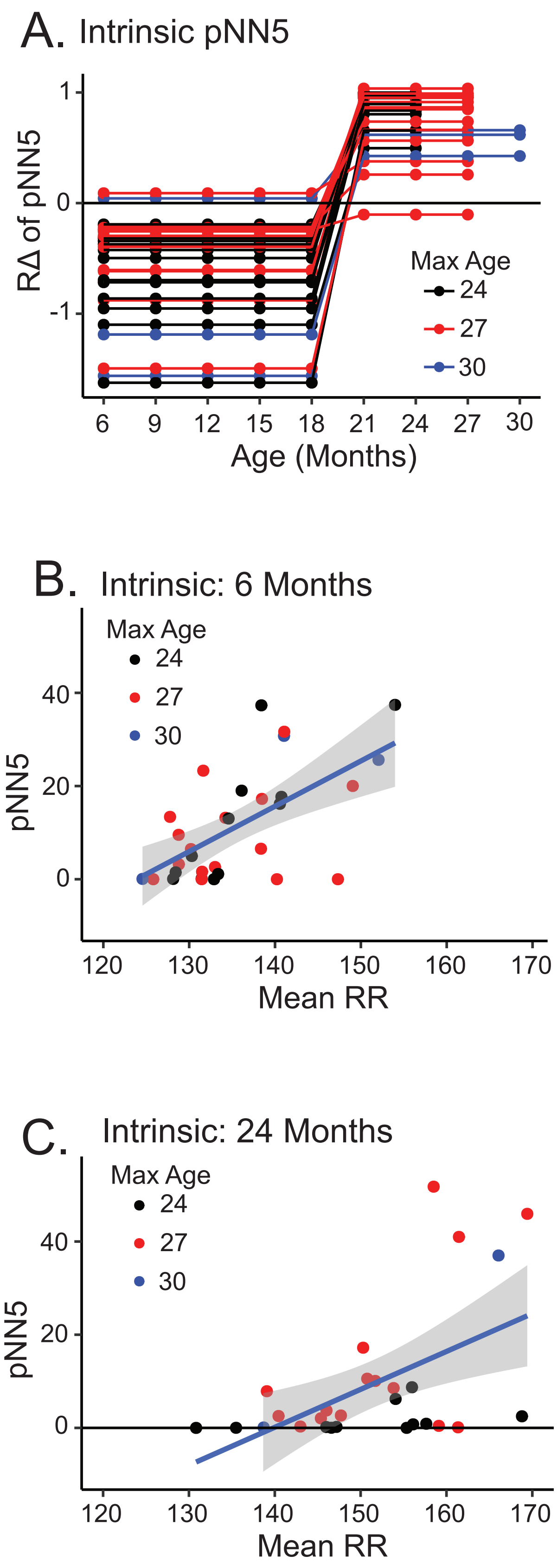

Figure S1. Mouse-specific RΔ for intrinsic pNN5, an index of long sinus pauses, in long-lived mice for the (A) RΔ, (B) intrinsic at 6 months of age, and (C) intrinsic at 24 months of age.

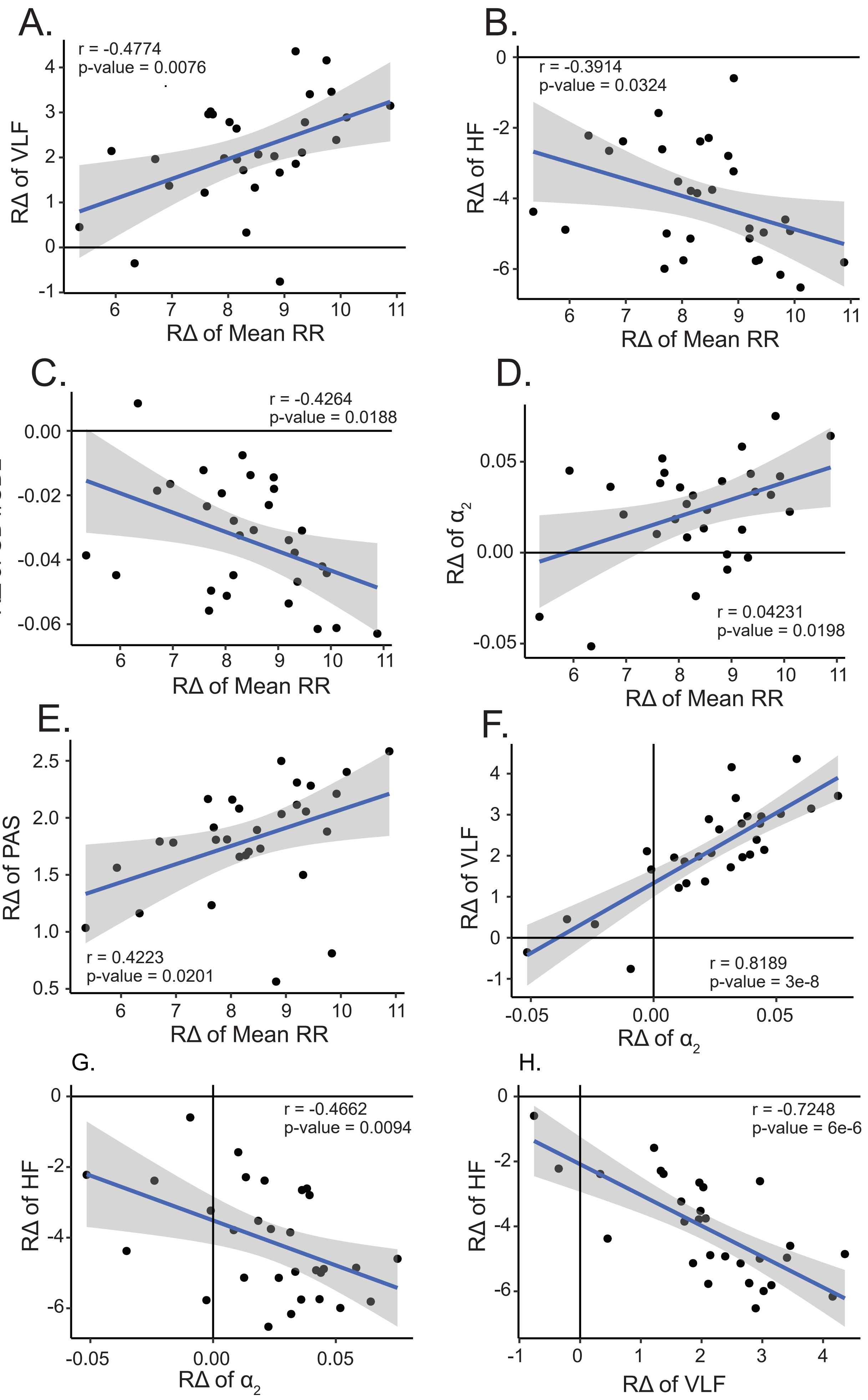

Figure S2. Mouse-specific intrinsic rΔ in long-lived mice

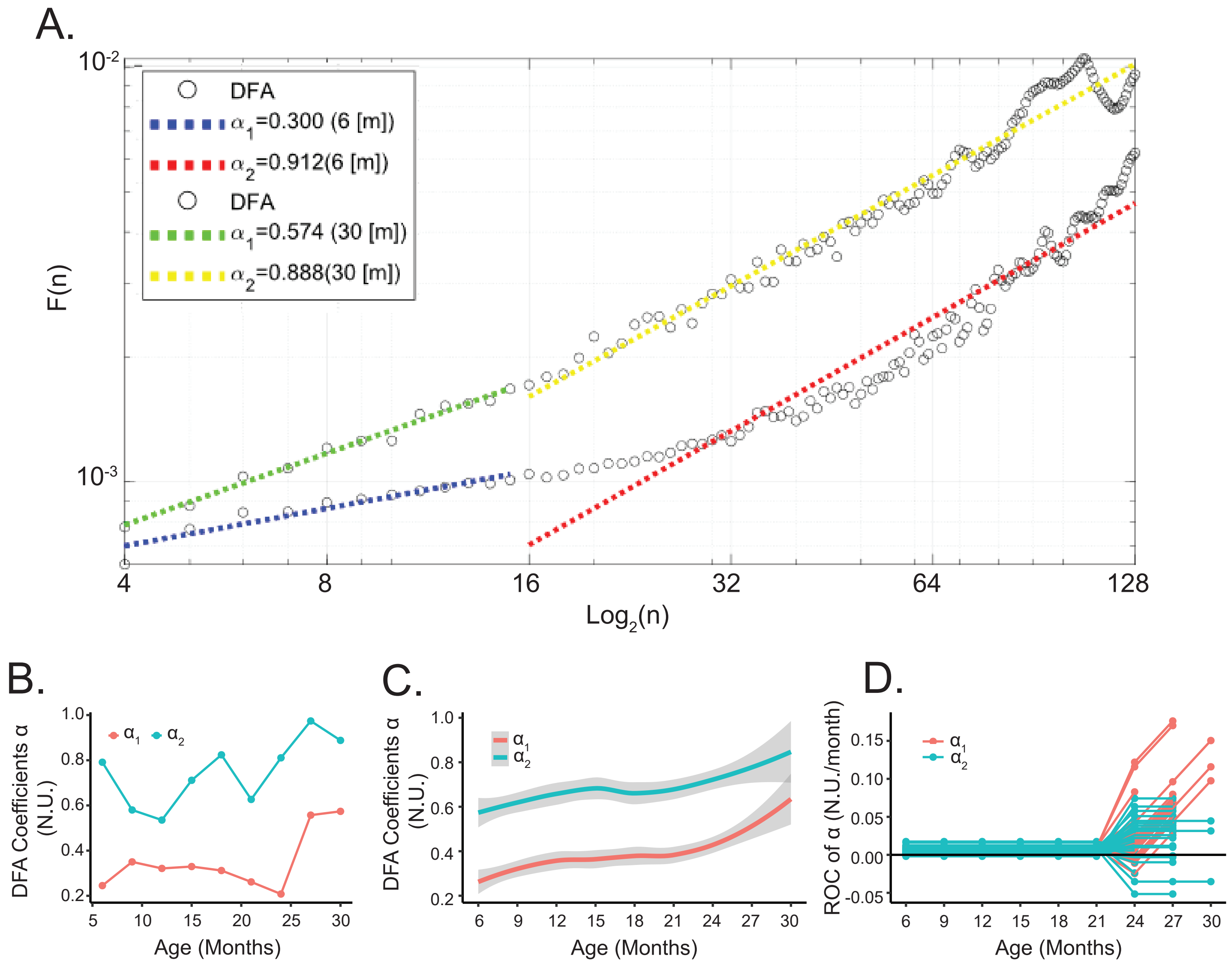

Figure S3. A. Detrended Fluctuation Analysis (DFA) illustrating intrinsic α1 and α2 at 6 and 30 months for mouse ZA1730.
B. Trajectories of Intrinsic α1 and α2 in mouse ZA1730.
C. Average loess smooth curves of intrinsic α1 and α2.
D. Mouse-specific rates of change of intrinsic α1 and α2 in long-lived mice.

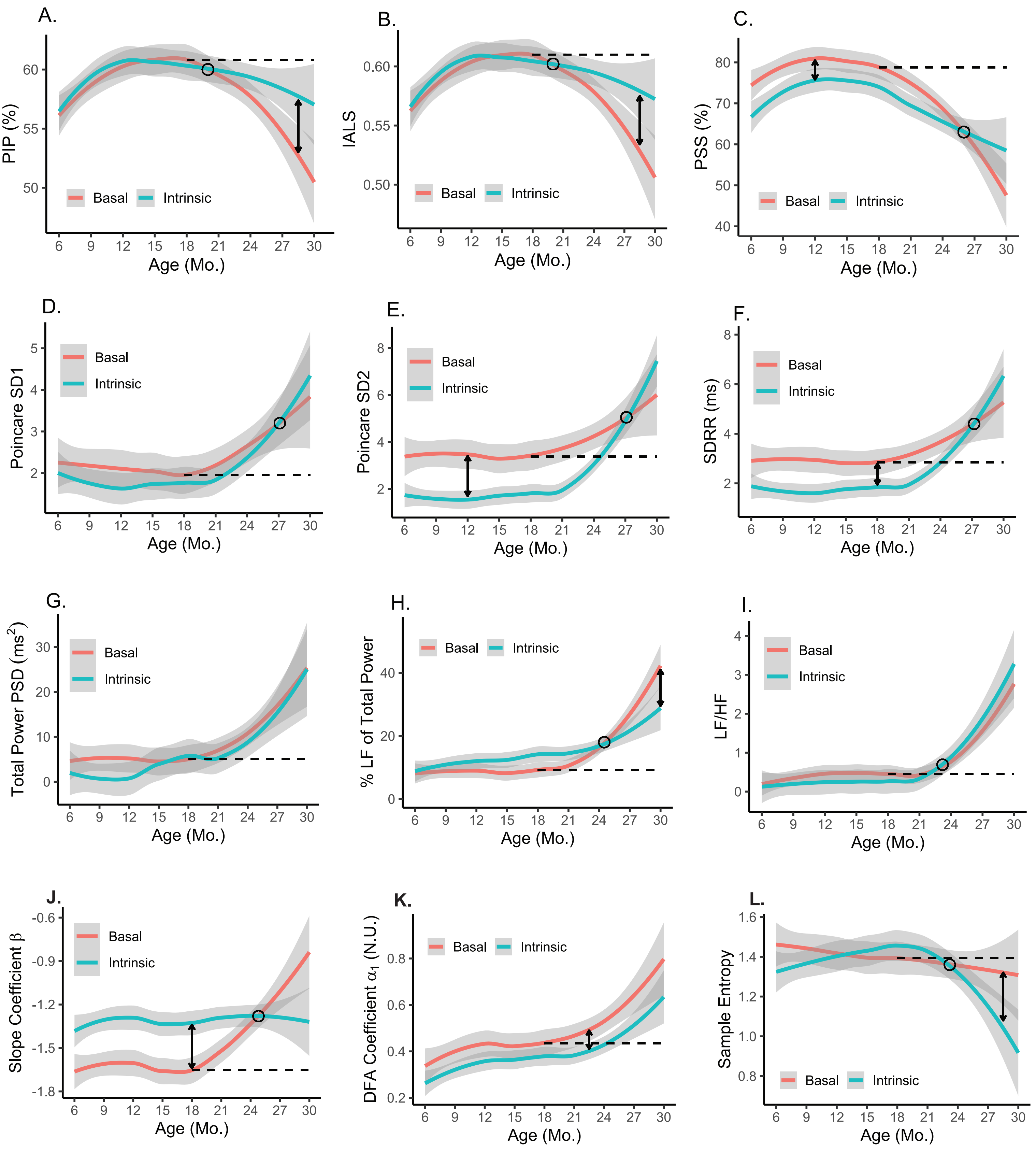

Figure S4. Average loess smooth curves of basal and intrinsic: A. PIP, B. IALS, C. PSS, D. SD1, E. SD2, F. SDRR, G. Total Power PSD, H. % LF of Total Power, I. LF/HF, J. Slope coefficient, β, K. DFA coefficient α1, and L. Sample entropy.

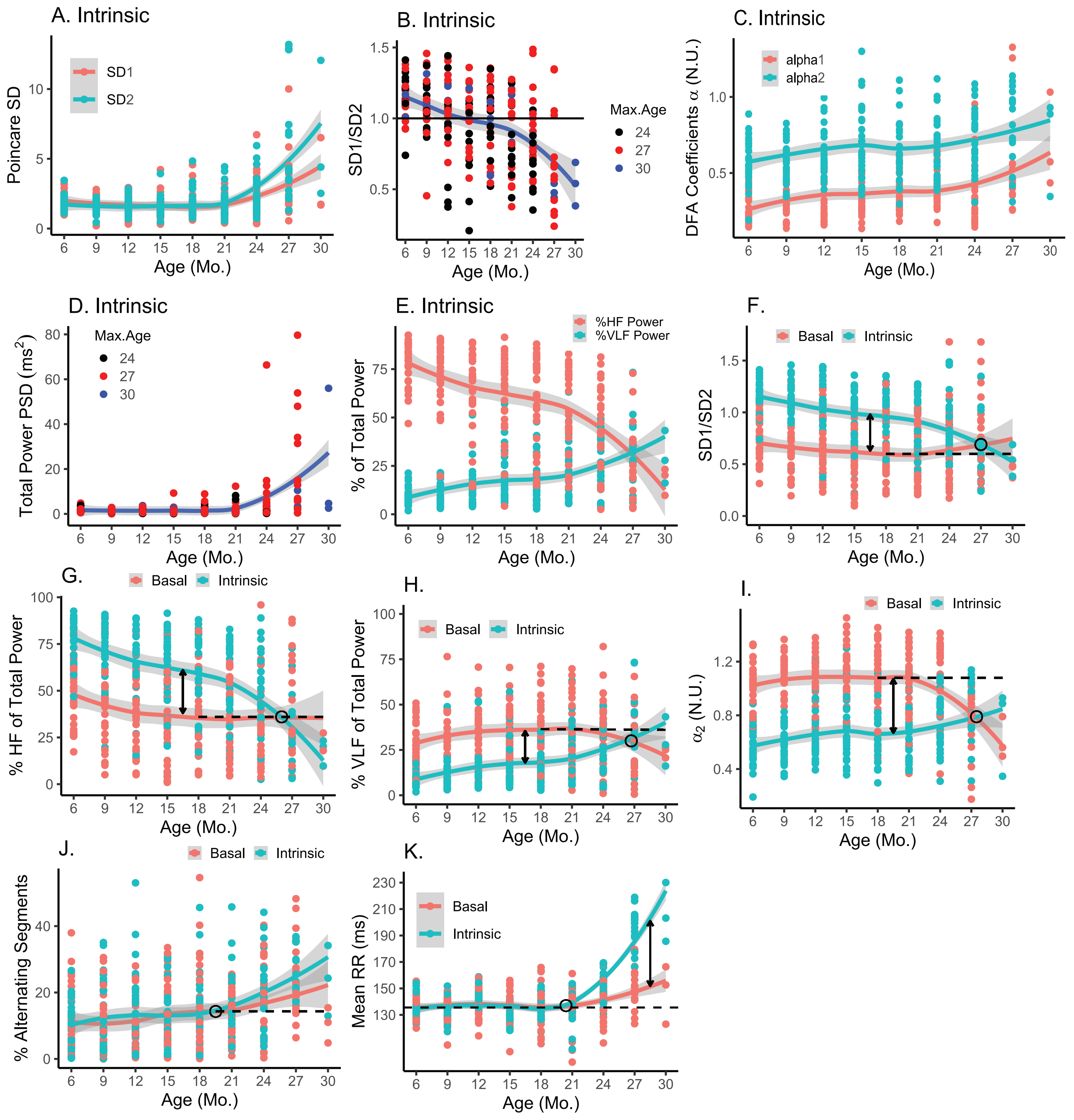

Figure S5. A. Intrinsic SD1 and SD2 with average loess smooth curves and the observed measurements, B. Intrinsic SD1/SD2 with average loess smooth curve and the observed measurements, C. Intrinsic α1 and α2 with average loess smooth curves and the observed measurements, D. B. Intrinsic Total Power PSD with average loess smooth curve and the observed measurements, E. Intrinsic %HF and %VLF of Total Power with average loess smooth curves and the the observed measurements, F. Basal and Intrinsic SD1/SD2 with average loess smooth curve and the observed measurements, G. Basal and Intrinsic %HF of Total Power with average loess smooth curve and the observed measurements, H. Basal and Intrinsic %VLF of Total Power with average loess smooth curve and the observed measurements, I. Basal and Intrinsic α2 with average loess smooth curve and the observed measurements, J. Basal and Intrinsic PAS with average loess smooth curve and the observed measurements, and K. Basal and Intrinsic Mean RR with average loess smooth curve and the observed measurements.

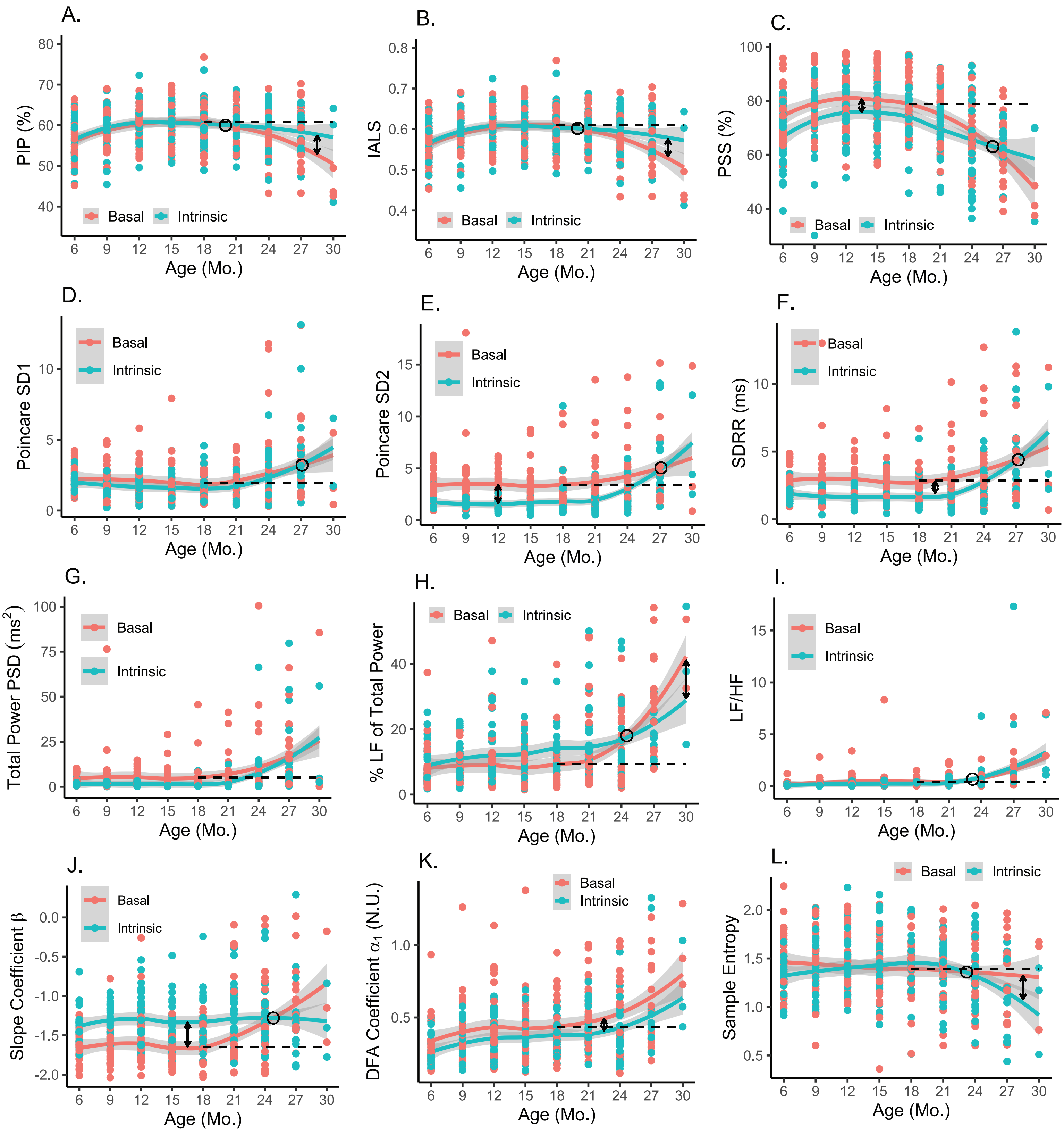

Figure S6. Basal and Intrinsic data with average loess smooth curve and the observed measurements for: A. PIP, B. IALS, C. PSS, D. SD1, E. SD2, F. SDRR, G. Total Power PSD, H. % LF of Total Power, I. LF/HF, J. Slope coefficient, β, K. DFA coefficient α1, and L. Sample entropy.

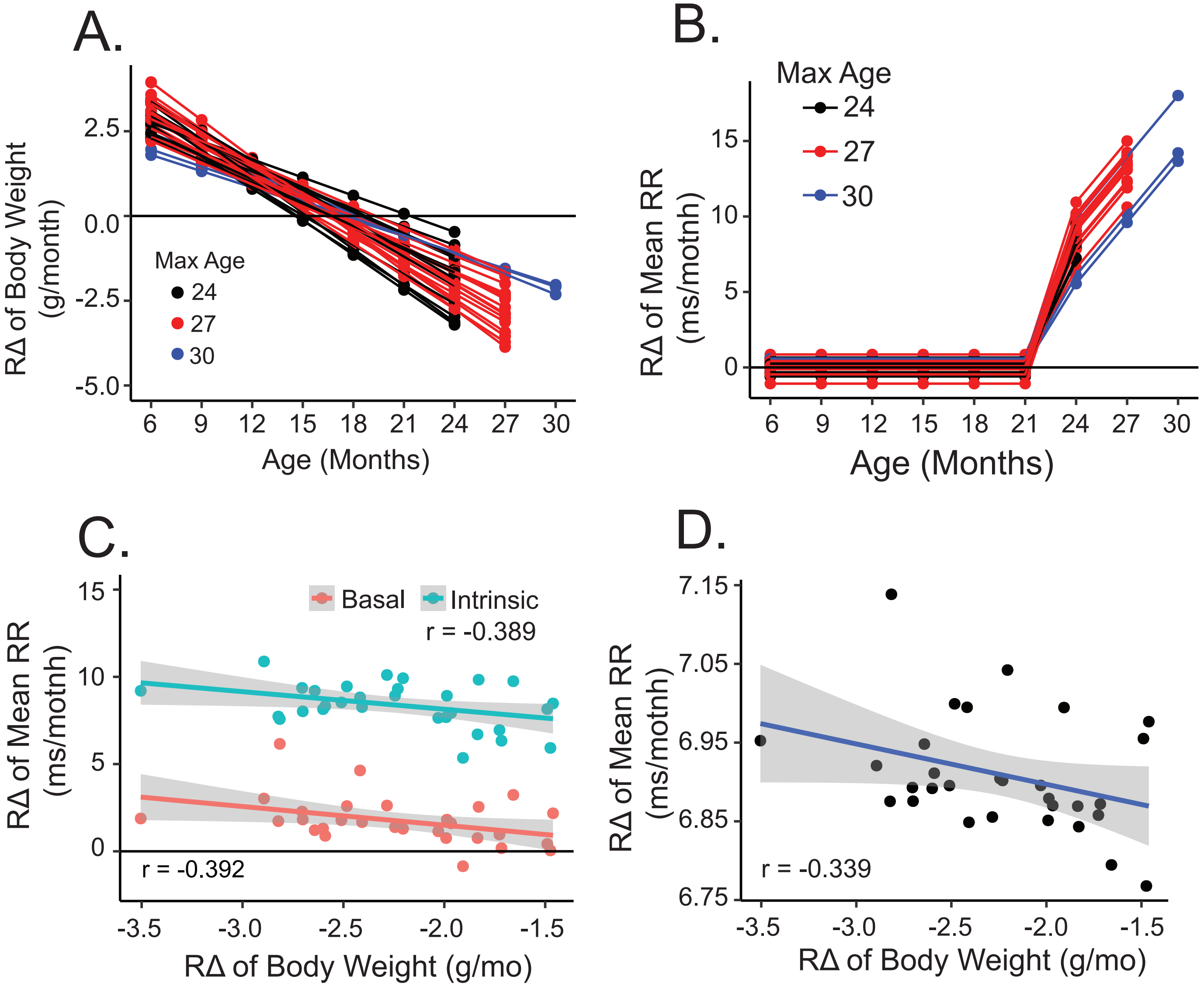

Figure S7. Mouse-specific rΔ in long-lived mice for:
A. Body Weight, and B. Intrinsic Mean RR.

Table S4. Standard deviation among mice in rates of change of body weight, mean RR, and RR interval variability parameters. Early period is at 6 months. Late period is at 24 months. A test of equality of variances from paired data is obtained using the Pitman Morgan test. The ratio is SD_{Late}/SD_{Early}.

	Basal				Intrinsic				Autonomic Effect			
	Early	Late	p-value	Ratio	Early	Late	p-value	Ratio	Early	Late	p-value	Ratio
BW	0.2559	0.4932	0.0005	1.93								
Mean.RR	0.3007	1.3475	3.5E-13	4.48	0.3853	1.2798	1.0E-08	3.32	0.4712	0.07471	2.2E-16	0.16
SDRatio	0.002856	0.01750	1.6E-15	6.13	0.006449	0.01801	3.8E-09	2.79	0.007809	0.00017	< 2.2e-16	0.02
VLF	0.4142	0.2097	1.0E-06	0.51	0.3670	1.1834	7.1E-10	3.23	0.4537	0.1607	4.4E-08	0.35
HF	0.3866	0.6242	0.0131	1.61	0.5264	1.5475	1.6E-09	2.94	0.5068	0.4845	0.7590	0.96
alpha2	0.003831	0.00097	3.4E-12	0.25	0.00705	0.02836	5.2E-13	4.02	0.005434	0.01225	8.3E-07	2.25
PAS	0.2518	0.9935	1.2E-10	3.95	0.1353	0.4815	2.9E-12	3.56	0.1788	1.1216	8.9E-16	6.27

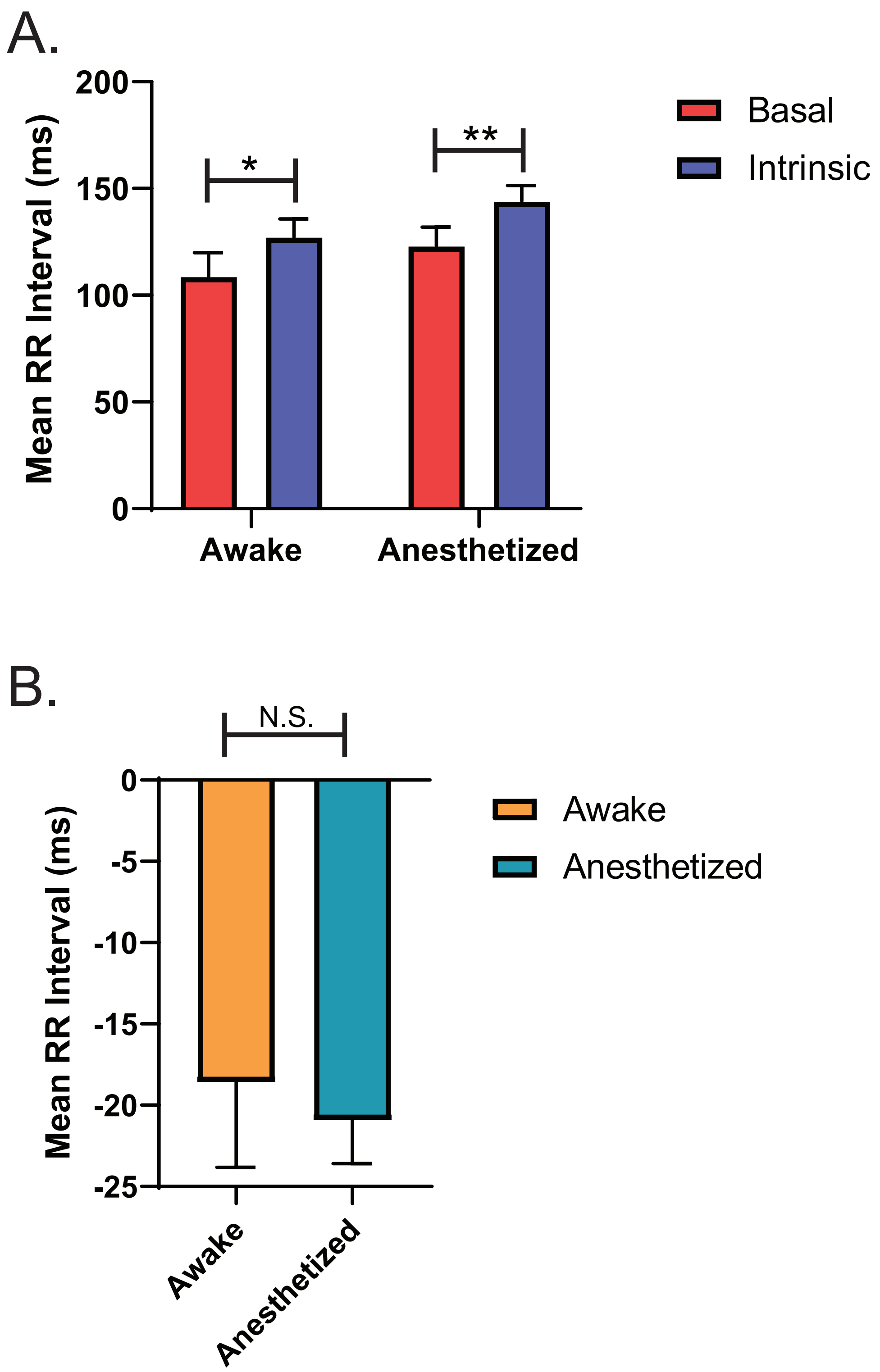

Figure S8. A. Mean RR interval in the presence and absence of autonomic blockade in the awake vs. anesthetized state. B. Autonomic input (the difference between basal and intrinsic) mean RR interval.
